# Integrin α8‐Mediated Pericyte Morphogenesis Controls Blood‐Brain Barrier Integrity

**DOI:** 10.1002/advs.202415374

**Published:** 2025-10-14

**Authors:** Chang‐Xiong Gong, Lin‐Lin Hu, Ling Jiang, Pei‐Xia Shi, Shuang Zhang, Yuan Zhao, Bing‐Qiao Wang, Xiao‐Feng Cheng, Cheng‐Kang He, Sen Lin, Yue Dai, Zhao‐You Meng, Chen‐Hao Zhao, Bao‐Liang Sun, Qi Xie, Fang‐Fei Li, Qing‐Wu Yang

**Affiliations:** ^1^ Department of Neurology, Xinqiao Hospital The Army Medical University Chongqing 400037 China; ^2^ Chongqing Institute for Brain and Intelligence Guangyang Bay Laboratory Chongqing 400064 China; ^3^ Department of Neurology, The Second Affiliated Hospital, Key Laboratory of Cerebral Microcirculation in Universities of Shandong Shandong First Medical University & Shandong Academy of Medical Sciences Taian Shandong 271000 China

**Keywords:** BBB, ITGA8, morphology, pericyte

## Abstract

Pericytes exhibits distinct morphological features on microvessels during various stages of brain‐blood barrier (BBB) development and neurological disorders. However, the underlying mechanisms by which pericyte morphology influences BBB integrity are not yet fully understood. Integrin α8 (*ITGA8*) is prominently expressed in mature pericytes, which tightly associate with endothelial cells during BBB maturation and post‐ischemic vascular remodeling. *ITGA8*‐deficient mice shows the disrupted pericyte morphology, including shortened processes and reduced vascular coverage, leads to compromised BBB integrity. Mechanistically, *ITGA8* regulates pericyte morphology via the Rho‐ROCK signaling, subsequently impacting BBB function through TGF‐β1 activation, which underscores its role in cytoskeletal organization and microvascular stability. In an ischemic stroke model, *ITGA8* deficiency results in increased BBB permeability, reduced pericyte coverage, and worsened neurological recovery. Conversely, adeno‐associated virus (AAV)‐mediated *ITGA8* overexpression restores pericyte morphology and improveS post‐stroke neurological outcomes. *ITGA8* is identified as a key regulator of pericyte morphology and function, which are essential for maintaining BBB integrity, thereby highlighting the therapeutic potential of targeting *ITGA8* for BBB repair and neurovascular recovery.

## Graphical abstract

This study highlights that *ITGA8* impacts pericyte morphology and function, both crucial for BBB integrity, via RhoA/ROCK signaling, thus influencing TGF‐β1 activation through cytoskeletal tension and ECM interactions. Notably, in post‐ischemic recovery models, *ITGA8* deficiency exacerbates vascular dysfunction by impairing pericyte‐mediated BBB reconstruction, whereas therapeutic *ITGA8* overexpression enhances vascular stabilization and recovery.

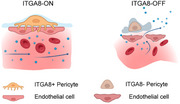

## 1 Introduction

Maintaining the integrity of the blood‐brain barrier (BBB) is critical for neural function and homeostasis.^[^ ^1^, ^2^, ^3^ ^]^ Disruption of BBB integrity is associated with severe neurological conditions, including stroke, multiple sclerosis, and Alzheimer's disease, underscoring the imperative to elucidate molecular mechanisms regulating its structural and functional stability.^[^ ^3^, ^4^, ^5^ ^]^

Brain vascular pericytes are key players in maintaining BBB integrity.^[^ ^6^, ^7^ ^]^ These mural cells, embedded within the basement membrane of capillaries and microvessels, provide structural support, regulate blood flow, and facilitate endothelial cell function.^[^ ^8^, ^9^, ^10^ ^]^ Pericytes are characterized by their extensive cytoplasmic processes that wrap around endothelial cells, contributing to the formation of tight junctions and the regulation of vascular permeability.^[^ ^11^, ^12^ ^]^ Given their central role in the neurovascular unit, pericytes are crucial for the preservation of the BBB and overall brain homeostasis; yet the specific mechanisms by which they modulate BBB integrity remain largely unknown.

**Table 1:** Primary Antibodies for immunoblotting.

Antibody	Brand	Catalog number	Dilution
GAPDH Monoclonal Antibody	Proteintech	60004‐1	1:5000
Integrin α8 / *ITGA8* antibody	Santa	sc‐365798	1:500
Myosin Light Chain 2 (MLC) (D18E2) antibody	Cell Signaling Technology (CST)	8505	1:1000
Phospho‐Myosin Light Chain 2 (Thr18/Ser19) antibody (E2J8F)	CST	95777	1:1000
RhoA (67B9) antibody	CST	2117	1:1000
SMAD2/3 antibody	CST	3102	1:1000
p‐SMAD2/SMAD3 (Ser465, Ser467, Ser423, Ser425) polyclonal antibody	Invitrogen	PA5‐110155	1:500
PI3 Kinase p85 (19H8) Rabbit mAb	CST	4257	1:1000
Phospho‐PI3 Kinase p85 (Tyr458)/p55 (Tyr199) Antibody	CST	4228	1:1000
Akt (pan) (C67E7) Rabbit mAb	CST	4691	1:1000
Phospho‐Akt (Ser473) (D9E) XP® Rabbit mAb	CST	4060	1:1000
p38 MAPK (D13E1) XP® Rabbit mAb	CST	8690	1:1000
Phospho‐p38 MAPK (Thr180/Tyr182) (D3F9) XP® Rabbit mAb	CST	4511	1:1000
SAPK/JNK Antibody	CST	9252	1:1000
Phospho‐SAPK/JNK (Thr183/Tyr185) (81E11) Rabbit mAb	CST	4668	1:1000
p44/42 MAPK (Erk1/2) (137F5) Rabbit mAb	CST	4695	1:1000
Phospho‐p44/42 MAPK (Erk1/2) (Thr202/Tyr204) (D13.14.4E) XP® Rabbit mAb	CST	4370	1:1000
Jak1 (6G4) Rabbit mAb	CST	3344	1:1000
Jak2 (D2E12) XP® Rabbit mAb	CST	3230	1:1000
Phospho‐Jak1 (Tyr1034/1035)/Jak2 (Tyr1007/1008) (E9Y7V) Mouse mAb	CST	66245	1:1000
Stat1 Antibody	CST	9172	1:1000
Phospho‐Stat1 (Tyr701) (58D6) Rabbit mAb	CST	9167	1:1000
Stat3 (124H6) Mouse mAb	CST	9139	1:1000
Phospho‐Stat3 (Tyr705) (M9C6) Mouse mAb	CST	4113	1:1000
Anti‐Integrin beta 1 antibody	Abcam	ab179471	1:2000

**Table 2:** Primary Antibodies for immunostaining.

Antibody	Brand	Catalog number	Dilution
Goat polyclonal anti‐Integrin alpha 8	RD R&D Systems (RD)	AF4076	1:500
Rat monoclonal anti‐CD31	BD Biosciences	550274	1:200
Goat polyclonal anti‐CD31	RD	AF3628	1:300
Rabbit monoclonal anti‐PDGFR‐β	CST	28E1	1:150
Rabbit monoclonal anti‐CD13	CST	D6V1W	1:200
Rabbit monoclonal anti‐Claudin5	Abcam	ab131259	1:150
Rabbit monoclonal anti‐Occludin	Abcam	ab216327	1:150
Rabbit monoclonal anti‐ZO1	Abcam	ab221547	1:200
Mouse monoclonal anti‐N‐cadherin	Santa	sc‐8424	1:100
Rabbit polyclonal anti‐Laminin	Abcam	ab11575	1:100
Rabbit polyclonal anti‐Collagen IV	Abcam	ab6586	1:500
Rabbit polyclonal anti‐alpha smooth muscle Actin	Abcam	ab5694	1:100
Rabbit monoclonal anti‐MAP2	Abcam	ab183830	1:500
Mouse monoclonal anti‐Bassoon	Abcam	ab82958	1:500
Goat polyclonal anti‐EphB4	RD	AF446	1:50
Rabbit monoclonal anti‐Glucose Transporter GLUT1	Abcam	ab115730	1:200
Rabbit polyclonal anti‐Homer1	Synaptic System	160003	1:500

**Table 3:** Secondary Antibodies for immunoblotting.

Antibody	Brand	Catalog number	Dilution
(HRP)‐conjugated goat anti‐mouse IgG(H+L)	Beyotime Biotechnology	A0216	1:3000
(HRP)‐conjugated goat anti‐rabbit IgG(H+L)	Beyotime Biotechnology	A0208	1:3000

**Table 4:** Secondary Antibodies for immunostaining.

Antibody	Brand	Catalog number	Dilution
Goat anti‐Mouse IgG (H+L) Highly Cross‐Adsorbed Secondary Antibody, Alexa Fluor™ Plus 555	Invitrogen	A32727	1:1000
Goat anti‐Rabbit IgG (H+L) Highly Cross‐Adsorbed Secondary Antibody, Alexa Fluor™	Invitrogen	A32732	1:1000
Goat anti‐Rat IgG (H+L) Cross‐Adsorbed Secondary Antibody, Alexa Fluor™ 647	Invitrogen	A21247	1:1000
Goat anti‐Rabbit IgG (H+L) Cross‐Adsorbed Secondary Antibody, Alexa Fluor™ 647	Invitrogen	A21244	1:1000
Goat anti‐Mouse IgG (H+L) Cross‐Adsorbed Secondary Antibody, Alexa Fluor™ 647	Invitrogen	A21235	1:1000
Goat anti‐Mouse IgG (H+L) Highly Cross‐Adsorbed Secondary Antibody, Alexa Fluor™ Plus 488	Invitrogen	A32723	1:1000
Goat anti‐Rabbit IgG (H+L) Highly Cross‐Adsorbed Secondary Antibody, Alexa Fluor™ Plus 488	Invitrogen	A32731	1:1000
Goat anti‐Mouse IgG (H+L) Highly Cross‐Adsorbed Secondary Antibody, Alexa Fluor™ Plus 555	Invitrogen	A32727	1:1000
Goat anti‐Rabbit IgG (H+L) Highly Cross‐Adsorbed Secondary Antibody, Alexa Fluor™ Plus 555	Invitrogen	A32732	1:1000
Donkey anti‐Goat IgG (H+L) Highly Cross‐Adsorbed Secondary Antibody, Alexa Fluor™ Plus 647	Invitrogen	A32849	1:1000
Donkey anti‐Rabbit IgG (H+L) Highly Cross‐Adsorbed Secondary Antibody, Alexa Fluor™ Plus 555	Invitrogen	A32794	1:1000

**Table 5:** Primers for genotype.

Mouse Strains	Prime sequence	Band Size
*ITGA8*‐P2A‐iCre	F1: GGCTGGACCAATGTGAACATTG; R1: TCATATTGGGCTGACCTAGACAGC	WT:0bp KO:414bp
	F2: ATAGAGCAAGACCTCCTCAGGATGA R2: TCATATTGGGCTGACCTAGACAGC	WT: 375bp KO: 1514bp
B6/JGpt‐H11^em1Cin(CAG‐LoxP‐ZsGreen‐Stop‐LoxP‐tdTomato)^	F1: TACGGCATGGACGAGCTGTAC R1: CCAACCTTTGTTCATGGCAG	WT: 0bp Targeted: 333bp
	F2: CAGCAAAACCTGGCTGTGGATC R2: ATGAGCCACCATGTGGGTGTC	WT: 412bp Targeted: 0bp
*Pdgfrb*‐P2A‐iCre	F1: TCTTCCCGCTCT CAAGTTCTAGC R1: GTTCTGGGAGGCAGAAGGGAGATTC	WT: 411bp Targeted: 1533bp
	F2: TCTTCCCGCTCTCAAGTTCTAGC R2: GCACACAGACA GGAGCATCTTC	Targeted: 595bp
*Pdgfrb*‐P2A‐ CreER^T2^	F: GAACTGTCACCGGGAGGA R: AGGCAA ATTTTGGTGTACGG	Targeted: 400bp
	Internal Positive Control F: CAA ATGTTGCTTGTCTGGTG Internal Positive Control R: GTCAGTC GAGTGCACAGTTT	Control: 200bp
*ITGA8^fl/fl^*	F: TCCTTGTCT TCCTGTGTATTTGAC R: TGTCTGAGA AGATTCAGCAGTGGG	Targeted: 278bp WT: 244bp

The morphology of pericytes is intimately linked to the integrity of the BBB. During development and tissue growth, pericytes actively participate in vascular remodeling processes.^[^ ^13^, ^14^ ^]^ This process involves pericyte proliferation, elongation, and reorganization to align along the nascent vessels.^[^ ^14^, ^15^, ^16^ ^]^ In angiogenesis, which is the formation of new blood vessels from pre‐existing ones, pericytes detach from the parent vessel, migrate toward the angiogenic sprouts, and associate with newly forming endothelial cells to support vessel formation and stabilization.^[^ ^17^, ^18^ ^]^ Proper pericyte function and morphology, including their ability to maintain close associations with endothelial cells, are essential for BBB stability.^[^ ^19^ ^]^ Clinically, such pericyte morphological aberrations correlate with disease progression, making it imperative to understand the factors that regulate pericyte structure and coverage.^[^ ^20^, ^21^ ^]^

Multiple factors influence pericyte morphology and function, including extracellular matrix (ECM) components, growth factors, and integrins.^[^ ^22^, ^23^, ^24^ ^]^ Platelet‐derived growth factor (PDGF) and transforming growth factor‐β (TGF‐β) are key mediators of pericyte recruitment, differentiation, and migration.^[^ ^25^, ^26^ ^]^ Integrins, a family of cell surface receptors, mediate cell‐ECM adhesion and signaling, also playing a crucial role in the regulation of the cytoskeleton and cellular behavior.^[^ ^27^, ^28^, ^29^ ^]^ However, the specific signaling pathways and molecular mechanisms involved in pericyte structural regulation to maintain BBB integrity remain unknown.

Here, we identified Integrin α8 (*ITGA8*), a pericyte‐enriched integrin subunit, emerges as a critical regulator of BBB integrity through its control of pericyte morphogenesis. During BBB maturation and post‐ischemic vascular remodeling, *ITGA8* is predominantly localized to functionally mature pericytes that exhibit hallmark features of barrier‐competent cells, including endothelial contact establishment and extensive vascular coverage. Conditional deletion of *ITGA8* in adult brain pericytes disrupted BBB integrity and neurological function, with mutant pericytes displaying morphological aberrations such as irregular cell shape, stunted processes, and disorganized cytoskeletal architecture. These structural defects correlated with diminished pericyte‐endothelial interactions, reduced vascular coverage, and elevated paracellular leakage. Additionally, our study demonstrates that *ITGA8* influences pericyte morphology and function through RhoA/ROCK signaling, thereby modulating the activation of TGF‐β1 signaling by cytoskeletal tension and ECM interactions. Notably, in post‐ischemic recovery models, *ITGA8* deficiency exacerbated vascular dysfunction by impairing pericyte‐mediated BBB reconstruction, whereas therapeutic *ITGA8* overexpression enhanced vascular stabilization and recovery. Collectively, our work highlights *ITGA8* as a novel regulator of pericyte morphogenesis and BBB integrity, and as a potential target for restoring BBB function in cerebrovascular disease.

## 2 Experimental Section

### 2.1 Mutant Mice and Inducible Genetic Modifications

All animal procedures were conducted in accordance with the guidelines approved by the Animal Ethics Committee of the Army Medical University (Third Military Medical University, Chongqing, China) (approval number: SCXK 2022‐0011), and in compliance with the ARRIVE (Animal Research: Reporting of In Vivo Experiments) guidelines.

The following mouse strains were used: *ITGA8^flox/flox^* (The Jackson laboratory, stock 015840); *Pdgfrb*‐P2A‐CreERT2 (The Jackson laboratory, stock 029684); B6/JGpt‐H11em1Cin (CAG‐LoxP‐ZsGreen‐Stop‐LoxP‐tdTomato) (H11‐tdTomato‐GFP) (GemPharmatech Co. Ltd, stock T006163); *Pdgfrb*‐iCre (BIOCYTOGEN, stock 110129); *ITGA8*‐P2A‐iCre (GemPharmatech co. Ltd, Chengdu, China).

*ITGA8*‐P2A‐iCre knock‐in mice were engineered to express Cre recombinase under the control of the endogenous Integrin alpha eight promoter/enhancer sequences, using the CRISPR/Cas9 technology (GemPharmatech co. Ltd, Chengdu, China). Initially, a guide RNA (gRNA) targeting a sequence near the insertion site (AAGACCCCAGAGGCGTGACT) was designed and synthesized in vitro. Subsequently, a donor vector containing the inserted fragment was also designed and constructed in vitro. Cas9 mRNA, gRNA, and the donor vector were co‐injected into zygotes. Thereafter, the zygotes were transferred into the oviduct of pseudopregnant ICR females at 0.5 dpc. In addition, F0 mice was birthed after ≈19–21 days of transplantation, all the offspring of ICR females (F0 mice) were identified by PCR and sequencing of tail DNA. Finally, crossing positive F0 mice with wildtype mouse to build up heterozygous mice. Genotyping Genomic DNA from the tail of 10‐day mice (F0) was extracted using alkaline lysis method. The genotype for *ITGA8*‐P2A‐iCre knock‐in allele was verified by PCR technology and direct sequencing. Primers designed by GemPharmatech Co. Ltd for genotype were listed in Table 5.

To generate the PDGFRβ+ cell *ITGA8* knockout, *Pdgfrb*‐P2A‐CreERT2 transgenic mice was bred with *Itga8^flox/flox^* mice, to obtain *Pdgfrb*‐P2A‐CreERT2; *ITGA8^flox/flox^* inducible conditional knockout (*Itga8*iPCKO) mice. *Itga8*iPCKO mice were also interbred with H11‐tdTomato‐GFP mice to obtain *Pdgfrb*‐P2A‐CreERT2; *ITGA8^flox/flox^*; H11‐tdTomato‐GFP mice. *Itga8*iPCKO or *Itga8^flox/flox^* (control) or *Pdgfrb*‐P2A‐CreERT2; *ITGA8^flox/flox^*; H11‐tdTomato‐GFP or *Pdgfrb*‐P2A‐CreERT2; *H11‐tdTomato‐GFP* (control) mice were treated with tamoxifen (MCE, HY‐13757A) (intraperitoneal injection of tamoxifen, 1 mg once a day for 10 days) at 7–8 weeks of age. Both males and females (in equal proportion) were used in this study. The genetic background for all mice was predominantly C57BL6, with a minimum of six backcross generations.

### 2.2 Human Brain Samples

Human brain sections were obtained from Chinese Brain Bank Center (CBBC) (Wuhan, China). All brain samples were collected following the standardized operational protocol established by the Medical Ethics Committee of South‐Central University for Nationalities (2022‐scuec‐039).

### 2.3 Laser Capture Microdissection and Microvessel Fraction Isolation

Under microscopic visualization, microvessels of interest were selectively targeted and captured using a laser beam, while the surrounding tissue was left untouched. The captured microvessels were collected into tubes containing lysis buffer for subsequent RNA isolation. Special care was taken to minimize contamination from non‐microvessel tissue during the microdissection process. Total RNA was isolated from the microvessel fractions using a commercially available RNA isolation kit (K0732, Thermo Scientific) according to the manufacturer's instructions. Data were normalized to internal control genes, and statistical analysis was conducted using appropriate methods.

### 2.4 Middle Cerebral Artery Occlusion (MCAO) and Reperfusion

MCAO surgery was performed as previously described.^[^ ^30^ ^]^ The mice (7–8 weeks old) were anesthetized by intraperitoneal administration of pentobarbital (75 mg kg^−1^ body weight). Focal I/R was achieved by a transient middle cerebral artery occlusion (MCAO) surgery. Briefly, following a midline cervical incision, the left common carotid artery (CCA), the left external carotid artery (ECA) and left pterygopalatine artery were isolated and ligated under an operating microscope. The internal carotid artery (ICA) was clipped at the peripheral site of the bifurcation of the ICA and the pterygopalatine artery with a small vascular clip. Thereafter, a 6‐0 silicone‐coated filament (Jialing [1800 model], Shanghai, China) was advanced through the ICA to the carotid bifurcation of the ICA and ECA. The filament was advanced until light resistance was felt, so that the distances from the filament tip to the ICA–pterygopalatine artery bifurcation and the ICA‐ECA bifurcation were slightly 6  and 9 mm, respectively. The filament was removed after 90‐min occlusion. Mice were carefully cared and left in the cages for the next 24 h. In the sham‐operated mice, these arteries were visualized but not disturbed.

### 2.5 Single Cell RNA Sequencing

Tissues dissociation was performed as previously described.^[^ ^31^ ^]^ Mice aged 7–8 weeks underwent either sham or MCAO surgery. Brain tissues from the infarcted hemisphere were then harvested from these mice on day 1, 3, and 7 after the procedure. The ischemic hemisphere and the ipsilateral hemisphere (sham ipsilateral brain) from three mice of the same group were randomly combined into one mixed sample, and then, tissues were dissected and minced using a scalpel for digestion with papain (Worthington, Biochemical Corporation, USA) containing 1% DNase for 25 min at 37 °C. The minced brain tissues were triturated gently 5‐times with a 5 mL pipette during the incubation process. Next, the mixture was centrifuged at 1000 rpm for 5 min at 4 °C. The supernatants were discarded and resuspended the cell pellets in 10 mL 30% percoll (Solaribio, P8370) in PBS. After centrifuging the cell suspensions at 800 g for 10 min, the myelin debris fraction floating on the surface was removed. The pelleted cells were resuspended with 1% FBS and was went through 70 µm filters to achieve single cell suspensions. After treatment with blood cell lysis buffer (Solaribio, R1010) for 5 min at 4 °C, all samples were washed and resuspended in 1% FBS. The single cell samples were counted by Countess II Automated Cell Counter using a hemocytometer with trypan blue. The cell viability was above 83%, and cells were loaded to BD Rhapsody system and subjected to single cell RNA sequencing. Single‐cell isolation in microwells with subsequent cell lysis and capture of the poly‐adenylated mRNAs with the barcoded, magnetic capture‐beads was performed according to the manufacturer's instructions. Beads were collected into a tube before reverse transcription. Then, each cDNA molecule was tagged on the 5′ end (that is, the 3′ end of mRNA transcript) with a unique molecular identifiers (UMIs) during cDNA synthesis, and cell barcode indicating its cell of origin. Whole transcriptome libraries were prepared using the BD Rhapsody single‐cell whole‐transcriptome amplification (WTA) workflow including random priming and extension (RPE), RPE amplification PCR, and WTA index PCR. The quantity and quality of sequencing libraries was detected using a High Sensitivity DNA assay (Thermo Fisher Scientific). The libraries were sequenced by NovaSeq 6000 (Illumina, San Diego, CA) on a 150‐bp paired‐end run. Fastq files were processed via the standard Rhapsody analysis pipeline (BD Biosciences) on the Seven Bridges application (BD Biosciences) according to the manufacturer's recommendations. Sample demultiplexing, read alignment to NCBI reference genome, quantification and initial quality control (QC) of microfluidics‐based sequencing dataset was analyzed using the Seurat R package version 3.15.1. The sequencing results were presented in the format of expression matrix. Cells contained over 200 expressed genes and mitochondria UMI rate below 10% passed the cell quality filtering, and mitochondria genes were removed in the expression table in each sample. Principal components analysis (PCA) was constructed on the basis of the scaled data with top 2000 high variable genes. Identification of cell clusters on tSNE (t‐distributed stochastic neighbor embedding) were performed. The known neuronal cell marker genes were used to annotate cell type. Differentially expressed genes of each cluster were identified using the FindAllMarkers function of Seurat. The enriched GO terms and KEGG pathways were identified using the online tool DAVID: https://david.ncifcrf.gov/.

### 2.6 Western Blot Analysis

Brain tissues or cells were homogenized in cold RIPA lysis buffer supplemented with complete protease inhibitor cocktail (Bimake, B142002) and phosSTOP phosphatase inhibitor (MCE, HY‐K0021, HY‐K0022). Protein samples were resolved by sodium dodecyl sulfate‐polyacrylamide gel electrophoresis (SDS‐PAGE) and transferred onto polyvinylidene fluoride membranes by electroblotting. Target proteins were detected by using specific primary antibodies. After incubation with horseradish peroxidase‐conjugated secondary antibodies, the blots were visualized with the enhanced chemiluminescence system from ProteinSimple (Azure, C300, USA). The band intensities were quantified and normalized to the GAPDH or β‐actin. The primary antibodies and second antibodies used for immunoblotting were listed in Tables 1, 3.

### 2.7 Cell Lines

Human brain primary pericytes (HBPCs) (ScienCell,1200), human brain primary endothelial cells (HBECs) (ScienCell, 1000), and human brain primary astrocytes (ScienCell, 1800) were obtained from ScienCellTM Research Laboratories. They were cultured in Pericyte Medium (ScienCell, 1201), Endothelial Cell Medium (ScienCell, 1001), and Astrocyte Medium (ScienCell, 1801) respectively. All the cells cultured in a humidified atmosphere of 5% CO_2_. The cell lines had been identified to be correct.

### 2.8 Immunofluorescence Staining

Mice were sacrificed with deep anesthesia by intraperitoneal administration of pentobarbital (75 mg kg^−1^ body weight) and perfused intracardially with phosphate‐buffered saline (PBS) followed by ice‐cold 4% paraformaldehyde (PFA). The brains were removed and post‐fixed with 4% PFA overnight at 4 °C. Brains were cryoprotected by consecutive immersions in 15 and 30% sucrose in PBS at 4 °C. Samples were then embedded in Tissue‐Tek OCT compound and frozen on dry ice. Brains were sectioned coronally at 30 µm using a cryostat microtome. Brain sections were incubated with primary antibodies at 4 °C overnight after 60 min blocking and permeabilization with 10% goat serum, 0.1% Triton X‐100 in PBS at room temperature. After primary antibody incubation, samples were washed with PBS for three times and incubated with Alexa Fluor 488‐ or Alexa Fluor 555‐ or Alexa Flour 647‐conjugated secondary antibodies (Thermo Fisher Scientific, MA, USA) for 1.5 h at RT. The antibody specificity and genuine target staining were validated by setting negative controls, which were incubated overnight with PBS, and stained with the secondary antibody. Nuclei were counterstained with DAPI. Brain sections were washed with PBS for four times before mounting them using fluorescence‐mounting medium. Fluorescent signals were detected by using a laser scanning confocal microscopy. The primary antibodies and second antibodies used for immunofluorescence staining were listed in Tables 2, 4. To ensure methodological rigor and minimize sampling bias, coronal brain sections from the same anatomical level were selected for immunofluorescence staining in both experimental and control groups. For each mouse (represented as a single data point in this study), three discontinuous coronal sections were analyzed. Within each section, five random fields of view (FOVs) in the peri‐infarct region or infarct core were imaged using confocal microscopy. Quantitative analysis was performed as follows: For each mouse, the total values from 15 FOVs (3 sections × 5 FOVs/section) were averaged to generate a single biological replicate value.

### 2.9 FITC‐Dextran Assay

FITC‐dextran assay was performed as previously.^[^ ^32^ ^]^ Mice were positioned on a heating‐plate for 10 min to dilated caudal vein. (FITC)‐conjugated dextran (2000‐kDa, 70‐kDa; Sigma‐Aldrich) (100 mg kg^−1^) was injected intravenously into the tail vein of mice using a 30‐gauge disposable needle, 3 min prior to sacrifice. The animals were anesthetized by intraperitoneal injection of pentobarbital (20 mg kg^−1^) and were sacrificed by cutting neck. Brains were removed and embed in OCT medium (Tissue‐Tek). After snap freeze in liquid nitrogen, the brains were sectioned and immunostained.

### 2.10 Normalized Coverage Ratio

The coverage of CD31+ endothelial cells by *ITGA8*+ and PDGFRβ+ pericytes was quantitatively assessed to understand their relative contributions to vascular coverage. Using ImageJ, the areas where ITGA8+ or PDGFRβ+ signals overlapped with CD31+ signals were identified and measured. This step quantified the coverage of endothelial cells by each pericyte subtype. To account for the difference in cell numbers between ITGA8+ and PDGFRβ+ pericytes and provide a fair comparison, a normalization step was included. This involved dividing the area of endothelial coverage by the respective pericyte subtype by the total number of cells of that subtype in the same CD31+ area. The normalized coverage ratios of ITGA8+ and PDGFRβ+ pericytes were statistically analyzed to determine significant differences in their contributions to endothelial cell coverage.

### 2.11 Fluoro‐Jade B (FJB) Staining

FJB was used to stain the degenerating neurons.^[^ ^33^ ^]^ According to the Fluoro‐Jade B POWDER Stain Reagent (Biosensis, TR‐150‐FJB) product instruction, 4% PFA frozen brain sections on the gelatin‐coated slides were dried at 50–60 °C for 40 min, and then placed in 70% ethanol for 5 min, followed by three washes in distilled water for 2 min each rinse. Brain sections were oxidized by incubating in a solution of 0.06% potassium permanganate solution for 10 min. To halt staining, rinse slides for 2 min in fresh distilled water. Brain sections were subsequently stained in FJB work solution (0.0004% working solution in 0.1% acetic acid) for 10 min. The slides were then washed with distilled water for three times for each and dried overnight at room temperature. For the motor cortex and hippocampus CA1, six 20×fields/animal were collected/animal (*n* = 4) respectively.

### 2.12 Golgi Staining

Whole brains were dissected into 2–3 mm, immersed in 20 mL Golgi dye solution (Servicebio, G1069‐1), and stored in the dark at room temperature. After 14‐days, the brains were washed by distilled water for three times. 60 µm coronal slices were cut at room temperature with a Vibratome (Leica, VT1000S) and transferred onto 1% gelatin‐coated slides (Servicebio, G6012‐1) for staining. After immersion cleaning in distilled water for 5 min, the slides were immersed in stronger ammonia water for 10 min. After washed by distilled water for two times, the slides were immersed in acid hardening fixer (Servicebio, G1069‐2) in the dark room for 45 min. The slices were cleared in distilled water and cover slipped with neutral balsam mounting medium. All images were acquired with a tissue slice scanner (3DHISTECH, Pannoramic MIDI) and analyzed with Fiji.

### 2.13 Overexpression of *ITGA8* in Mice Brain

AAV‐PR capsid construction was consistent with previous studies^[^ ^34^ ^]^ and was purchased from GeneChem (Shanghai, China). The full‐length cDNA of *ITGA8* was inserted into AAV‐PR to create an *ITGA8* overexpression AAV‐PR vector. Subsequently, 6‐week‐old *ITGA8*iPCKO mice were injected with either AAV‐PR‐*ITGA8* or AAV‐PR vectors in a volume of 100 µL via the tail vein. The viral titer was 2 × 10^12^ genomes per mL. 20‐days later, these mice underwent MCAO surgery.

### 2.14 Morris Water‐Maze

The spatial memory ability was tested Morris water maze test and started at 2 or 30 days after injection of tamoxifen for 10 days. Morris water maze test was operated as previously described.^[^ ^35^ ^]^ A circular tank (diameter 1.2 m, depth 0.4 m) was filled with opaque water (25 ± 1 °C, depth 0.3 m) and the escape platform (10  × 10 cm) was submerged 1 cm below the water level. The animals swim patterns were registered with EthoVision XT version 15 (Noldus, Wageningen, The Netherlands). Each mouse was trained 4‐times per day from the four quadrants of the pool for five consecutive days. Each mouse was allowed to search for the platform within 60 sec each time. The sixth day was the testing period, and the platform was removed from the pool. Each mouse was allowed to swim in the pool for 60 sec to explore the platform position. Each mouse's trajectory in the pool was recorded by water maze software for analysis. Escape latency, swim distance, travel across platform, time in target zone, probe time and path length were recorded for each trial.

### 2.15 Rotarod

The rotarod (Unibiolab, Beijing, China) was used to measure neuromuscular coordination and balance of mice at 2 or 30 days after injection of tamoxifen, which was reflected by the latency for a mouse to fall from a rotating beam with a ramping speed, starting at 5 rpm and accelerating to 45 rpm over 100 sec. All mice needed to be trained five times the day before test. Each mouse was placed individually on the beam and the latency until the mouse slid off the drum was recorded using a stopwatch. The rotarod test was repeated three times for each mouse.

### 2.16 Pole‐Climbing Test (PCT)

A cork ball of 2.5 cm in diameter was fixed on the top of the stick with a length of 50 cm and diameter of 1 cm. Placing the mouse at the top of the pole in a head upward position, then the time that the mice turned around on the ball and the time that the mice climbed the whole pole were recorded. All mice need to be trained five times to familiarize themselves with the whole process before testing. The trial was repeated three times and the average value was recorded. In each trial, the mouse had maximum 120 sec to finish the task. In case the mouse slid down the pole, a trial score of 60 sec was given. In case the mouse fell, a trial score of 120 sec was given. Mice were tested at 2 or 30 days after injection of tamoxifen.

### 2.17 CatWalk

Gait parameters were evaluated using the CatWalk10.6 system (Noldus, Wageningen, The Netherlands). All data analyses were performed with a pixel threshold value 525 arbitrary units. The animals crossed a horizontal glass runway equipped with a standard charge‐coupled device camera connected to a computer with the CatWalk software. Each mouse was placed on a glass walkway of CatWalk XT and allowed to run across it in an unforced manner. The footprints were recorded by a high‐speed video camera. The data obtained from at least three runs to traverse the walkway were recorded at one trial, and parameters such as regularity index, print length, maximum contact area, swing speed, and step cycle were measured.

### 2.18 Lentivirus Infection

HBPCs were infected with lentivirus carrying NC shRNA, ITGA8/ITGB1 shRNA, Mock shRNA, or ITGA8/ITGB1 cDNA full length (1 × 10^8^ TU/well for 6‐well plate). At 4–5 days after infection, cells were used for further functional analysis. All of these lentiviruses were obtained from HANBIO technology Co. Ltd. (Shanghai, China).

### 2.19 Scratch Wound Healing Assay

Seeding 5 × 10^5^ cells per well of a six‐well dish and allowing them to grow to 90–95% confluence within 1–2 days. Once the cells reached the desired confluency, cells were serum‐starved overnight using a serum‐free medium. 12 h later, all cells with mitomycin C (5 µg mL^−1^) were treated for 1 h to eliminate any potential effects of proliferation on wound closure. An ultrafine tip marker was used to draw a reference line on the exterior plastic surface of each well. Three separate wounds were made per well by scratching the monolayer culture using a sterile 200 µL micropipette tip. Cells were carefully rinsed with PBS three times to remove cell debris as well as to eliminate any remaining mitomycin C. RGD at 50 µm or SB431542 (an inhibitor of TGFβ signaling pathway) (HY‐10431, MCE) at 5 µm were added to the medium. A reference line was used to monitor the same region at 12 and 24 h, and the images of the same wound field were acquired. The migrated area was quantified in each image using ImageJ software.

### 2.20 Cell Adhesion Assay

Cell adhesion was evaluated using the cell‐matrix adhesion assay as previously described.^[^ ^36^ ^]^ Briefly, cells were plated in duplicate on a 24‐well plate and treated with either 50 µm RGD peptide or 5 µm SB431542 for 24 h. Following incubation, the cells were transferred to a plate coated with 150 µL of type I collagen (10 µg mL^−1^) and allowed to adhere for 60 min. Adherent cells were then fixed in ethanol, stained with 0.1% crystal violet, and fixed in 0.2% Triton‐100 for measurement at 550 nm using a microplate reader.

### 2.21 Pericyte‐Endothelial Cell Co‐Culture In Transwell

An in vitro co‐culture system of endothelial cells and pericytes was established to assess the effect of pericyte integrin β1 (ITGB1) on endothelial cell barrier integrity. For this system, transwell inserts (8 µm pore size, Corning) were used. Pericytes were seeded on the basal side of the transwell insert, while endothelial cells were seeded on the apical side. To set up the co‐culture system, the insert was first inverted, and 100 µL of a pericyte cell suspension (6 × 10^4^ cells in HBPC medium) was added to the basolateral side of the transwell insert. The plate was then returned to the incubator for 2–3 h. After this incubation period, the insert was reverted. Once the pericytes reached 90% confluency, 100 µL of an endothelial cell suspension (7.5 × 10^4^ cells in HBEC medium) was added to the apical compartment of the transwell insert. The cells were allowed to adhere for 5 h. Subsequently, the medium was topped up to 700 µL with HBEC medium, and the plate was returned to the incubator. When the endothelial cells formed a confluent monolayer, the barrier integrity was assessed using a Streptavidin‐horseradish peroxidase (HRP, R&D Systems) assay. Briefly, horseradish peroxidase (HRP) was added to the upper compartment and incubated for 5 min. The HRP was then reacted with 3,3′,5,5′‐tetramethylbenzidine (TMB) substrate for an additional 5 min. The amount of HRP that passed through the cell layer into the lower chamber was quantified by measuring the absorbance at 450 nm.

### 2.22 Pericyte‐Endothelial Cell Co‐Culture in Fibrinogen

The fibrin‐based 3D co‐culture model was selected to study HBEC–HBPC interactions due to its low background growth‐factor content and highly controllable parameters, which help minimize confounding variables in cell–cell interaction assays. This pericyte–endothelial co‐culture system was implemented according to a previously established method.^[^ ^37^ ^]^ Briefly, HBPC and HBEC were trypsinized and then resuspended in a solution containing 5 mg mL^−1^ fibrinogen (Sigma, F8630), supplemented with 10 µm Ilomastat (MCE, HY‐15768), and seeded at a density of 16 × 10^6^ cells mL^−1^ (HBEC) and 2 × 10^6^ cells mL^−1^ (HBPC). The cell mixture was then mixed with 2 U mL^−1^ thrombin (Sigma‐Aldrich, T4648) in a 1:1 (vol:vol) ratio. To prevent dye diffusion into the fibrin gel, covered the media side of the central HBEC/HBPC fibrin channel with a single layer of HBEC by adding 10^5^ HBEC and tilting the tip vertically. The chips were incubated in a humid chamber and supplied with fresh endothelial growth medium every two days. By day 8, the developed vessels exhibited signs of maturity, such as being well‐perfusable and showing reduced remodeling. RGD was added at a concentration of 50 µm or SB431542 at 5 µm to the medium for an additional two days. On day 10, cells were fixed by introducing 4% PFA into the medium channel for 6 h, and then used for immunostaining.

### 2.23 Enzyme‐Linked Immunosorbent Assay

The concentrations of activated transforming growth factor beta1 (TGFβ1) released in the culture medium were quantified with a TGFβ1 Emax ImmunoAssay System (Elabscience, E‐EL‐0162c) in accordance with the manufacturer's instructions. The TGFβ1 Emax ImmunoAssay was a sensitive and specific assay for detection of biologically active TGFβ1 in an antibody sandwich format. Only the bioactive TGFβ1 was immunoreactive and detectable by TGFβ1 monoclonal and polyclonal antibodies. Experiments were conducted three times and in triplicate for all samples and standards for the statistical analyses.

### 2.24 RhoA Activation Assay

GTP‐loaded RhoA levels were determined using a RhoA activation assay kit (CST, 8820) based on a pull‐down method. The assay involved binding GTP‐bound RhoA to glutathione resin via a GST‐linked binding protein, centrifuging to remove unbound proteins, and then eluting for Western blot analysis.

### 2.25 GTP Protease‐Linked Immunosorbent Assay (GLISA) Analyses

GLISA was conducted to quantify RhoA activation using a specific kit (Cytoskeleton, BK124, Denver, CO, USA). The procedure was carried out according to the manufacturer's guidelines to accurately assess RhoA activity levels.

### 2.26 Statistical Analysis

All data were showed as mean ± SD. Statistical differences were analyzed by using GraphPad Prism 7. In addition, one way ANOVA was employed to compare differences among groups and post hoc pairwise comparisons were performed using Tukey's HSD test to control the family‐wise error rate, as it was appropriate for balanced sample sizes and provided a balance between statistical power and Type I error control. The Student *t*‐test was used to compare the differences between groups. *p* < 0.05 was regarded as statistical significance.

## 3 Results

### 3.1 Expression of ITGA8 on the Microvessels during Development

**Figure 1:**
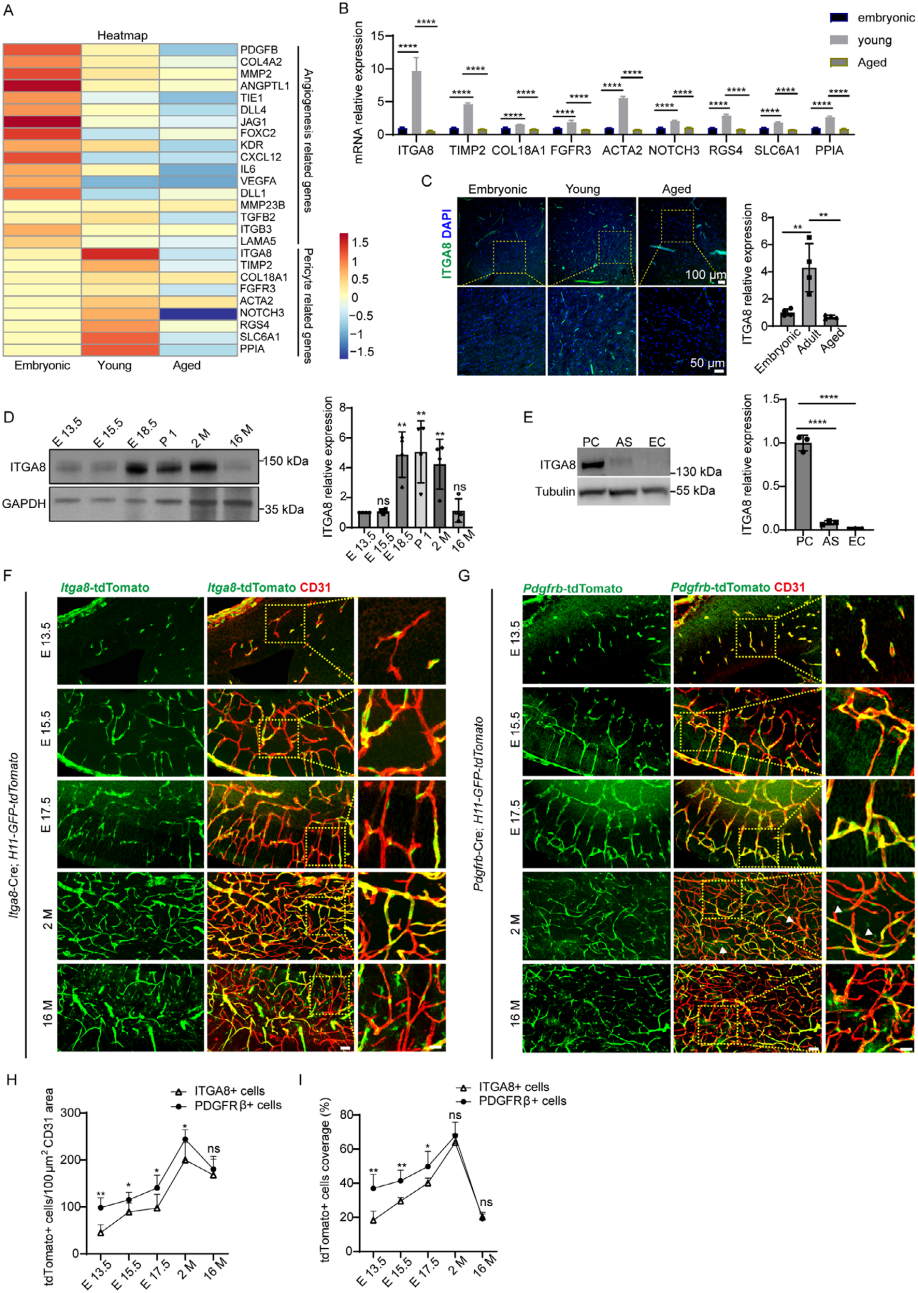
Expression Profile of *ITGA8* at Different Developmental Stage. A) RNA‐sequencing analysis reveals age‐dependent different genes in human brain microvessels (*n* = 3 samples). B) qPCR analysis confirms the age‐dependent modulation of pericyte related genes expression in human brain microvessels (*n* = 4 samples, all *p* < 0.0001). C) Representative immunostaining of *ITGA8* in human brain sections. Scale bars: 100 µm (upper), 50 µm (lower). *ITGA8* expression was quantified (*n* = 4 samples, *p* = 0.0028, 0.0015). D) Western blotting analysis of *ITGA8* protein levels at various developmental stages, normalized to sham controls. “E” denotes embryonic days, “P” denotes postnatal days, and “M” denotes age in months (*n* = 4 mice, *p* = 0.9999, 0.0022, 0.0014, 0.0098, and 0.9998). E) Western blotting indicates the predominant expression of *ITGA8* in human brain pericytes (PC) compared to astrocytes (AS) and endothelial cells (EC) (*n* = 3 cell samples, all *p* < 0.0001). F) Immunofluorescence showed the distinct temporal expression profile of *ITGA8*‐expressing cells during BBB development, with the right panels providing magnified views (scale bar: 15 µm) of the areas marked in the left panels (scale bar: 40 µm). G) The temporal expression profile of *Pdgfrb*‐expressing cells during BBB development, using immunofluorescence staining, and the right panels offer magnified views (scale bar: 15 µm) of the selected areas in the left panels (scale bar: 40 µm). Arrowheads point to scattered PDGFRβ+ cells within the brain parenchyma. H) Quantification of the density of tdTomato+ cells per 100 µm^2 of CD31 area in various brain regions, presenting a comparative analysis of tdTomato+ cell density between *ITGA8*+ cells and PDGFRβ+ cells across different ages. The number of tdTomato+ cells is normalized to the CD31 area, reflecting regional vascular density variations (*n* = 5 mice, *p* = 0.0022, 0.0467, 0.0428, 0.0460, and 0.5382). I) Quantification of the coverage of tdTomato+ cells on CD31+ vessels, comparing the coverage between *ITGA8*+ cells and PDGFRβ+ cells across various ages normalized to vascular area (*n* = 5 mice, *p* = 0.0028, 0.0035, 0.0468, 0.3657, and 0.6354). Data represent mean ± SEM. Significance notations: ns = not significant (*p* > 0.05), ^*^ *p* < 0.05, ^**^ *p* < 0.01, ^****^ *p* < 0.0001; Unpaired, 2‐tailed Student *t‐*test was used to compare groups in (H,I). Comparisons between multiple groups were made using one‐way ANOVA test followed by Tukey's HSD post hoc test in (B–D).

The BBB displays developmental stage‐dependent integrity dynamics.^[^ ^38^, ^39^ ^]^ To delineate microvascular transcriptional signatures across BBB maturation states, human brain tissues from three developmental stages (20‐week fetal, 18–45 years, > 70 years) were analyzed with ethical approval. Laser capture microdissection (LCM)‐purified microvessels underwent RNA sequencing, revealing age‐progressive regulation of angiogenesis pathways and pericyte‐related genes (Figure **1**A). *ITGA8* emerged as the most differentially expressed pericyte‐related gene in age‐stratified human microvessels, showing peak expression in young adults (18–45 years) followed by significant age‐related decline, as validated by qPCR analysis (Figure 1A,B). Immunostaining of human brain sections of cortex area showing the expression of *ITGA8* was highest in young adult brain (Figure 1C). In the mouse brain, *ITGA8* expression peaked at the mature stage of the BBB (Figure 1D). This age‐dependent modulation of *ITGA8* expression in human and mouse brain suggests a potential role for this integrin subunit in pericyte‐mediated BBB regulation. Western blot analyses established that *ITGA8* is predominantly expressed in human brain pericytes (HBPCs), in contrast to its expression in human astrocytes and endothelial cells (Figure 1E). Immunofluorescent staining of HBPCs showed the expression of *ITGA8* in PDGFRβ+ cells (Figure S1A, Supporting Information). These findings converge with single‐cell transcriptomics data^[^ ^40^ ^]^ demonstrated that *ITGA8* is highly expressed in smooth muscle cells and pericytes (Figure S1B, Supporting Information). Furthermore, we observed that *ITGA8* expression increases with brain development, peaking in the young adult brain at 24 and 25 years of age (Figure S1C, Supporting Information). Additionally, these data revealed that ITGA8's expression is reduced in low‐grade glioma, glioblastoma, and meningioma compared to the adult control brain (Figure S1D, Supporting Information). To spatially resolve *ITGA8*+ mural cells across neurodevelopment, we engineered *ITGA8‐*Cre; H11‐tdTomato‐GFP knock‐in mice (Figure S2A, Supporting Information) and established *Pdgfrb‐*Cre^[^ ^41^ ^]^; H11‐tdTomato‐GFP comparators (Figure S2B, Supporting Information), with subsequent analysis focused on the cortex area. Immunofluorescence staining confirmed that *ITGA8* expression in young adult mice (7–8 weeks old) was exclusively restricted to the PDGFRβ+ cell population (Figure S2C, Supporting Information). Furthermore, immunostaining demonstrated ITGA8 expression on mature vascular structures, including arterioles (α‐SMA+, >10 µm in diameter), capillaries (Glut1+, ≤10 µm in diameter), and venules (Ephrin B4+, >10 µm in diameter) (Figure S2D, Supporting Information), which aligns with previous findings.^[^ ^42^, ^43^ ^]^ Moreover, *ITGA8* expression was observed to be uniform throughout the whole brain (Data not shown). Our study delineates temporally regulated *ITGA8* expression during BBB development. *ITGA8* expression begins in the embryonic cerebral vasculature coinciding with BBB induction (Figure 1F). Its expression increases progressively throughout neurodevelopment, reaching peak levels in young adults when BBB integrity is fully mature. This pattern parallels the changes observed in PDGFRβ+ cells associated with vasculature (Figure 1F–I). In 2‐month‐old brains, *ITGA8*+ pericytes exhibited vascular maturation features: expansive vessel coverage via parallel‐aligned cytoplasmic projections, spindle‐shaped morphology, and tight endothelial interactions (Figure 1F). The spatiotemporal correlation of *ITGA8* expression with BBB development suggests its dual role in stabilizing vascular architecture and mediating endothelial‐pericyte crosstalk critical for BBB establishment and homeostasis.

Further comparative analysis of the *ITGA8*‐Cre; H11‐tdTomato‐GFP and *Pdgfrb*‐Cre; H11‐tdTomato‐GFP mouse models has elucidated distinct distribution patterns of *ITGA8*+ and PDGFRβ+ cells. Quantitative analysis showed PDGFRβ+ cell density exceeded *ITGA8*+ levels in embryonic and adult mice but became comparable in aged mice. Vascular coverage by PDGFRβ+ cells surpassed *ITGA8*+ cells during embryogenesis, while both showed equivalent coverage in adult and aged stages (Figure 1F–I). In addition, our findings indicate the exclusive expression of *ITGA8*, distinguishing them from another subset of PDGFRβ+ cells that solely express PDGFRβ without *ITGA8* co‐expression (PDGFRβ+ *ITGA8*−) (Figure 1F–G). The latter are dispersed throughout the brain parenchyma, in contrast to the perivascular positioning of ITGA8+ cells in adult mice (Figure 1F–G). Immunofluorescence staining in adult *Pdgfrb*‐Cre; H11‐tdTomato‐GFP brain sections corroborated these findings, revealing restricted *ITGA8* expression to perivascular PDGFRβ+ cells, whereas scattered parenchymal PDGFRβ+ populations remained *ITGA8*−(Figure S2C, Supporting Information). The proximity of *ITGA8+* pericytes to endothelial cells suggests they may play a key role in BBB maintenance.

### 3.2 Deletion of Pericyte ITGA8 Impairs Neural Function

**Figure 2:**
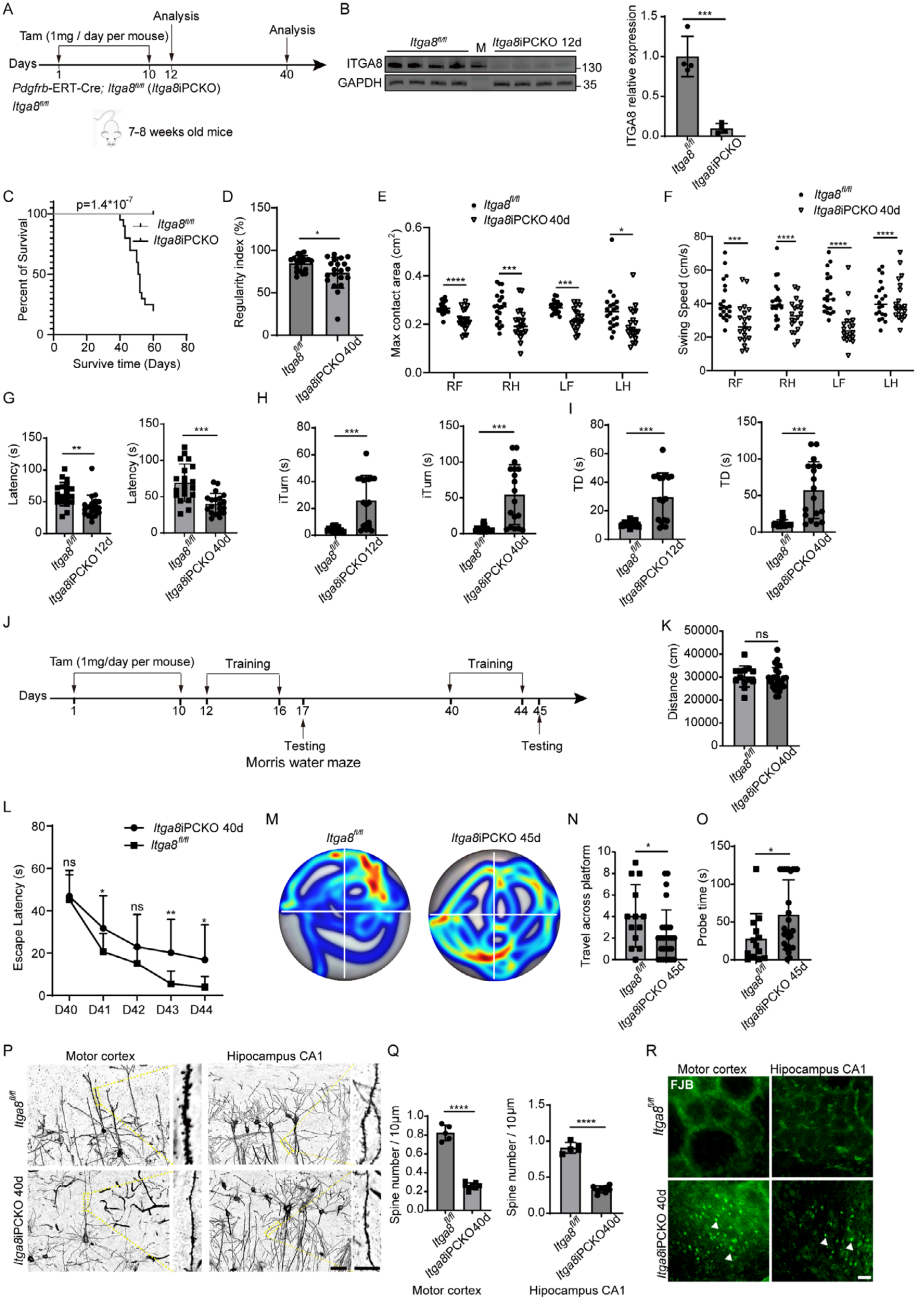
Deletion of Pericyte *Itga8* Impairs Neural Function. A) Schematic illustration of the experimental paradigm for conditional knockout of pericyte *ITGA8* using tamoxifen intraperitoneal injection, followed by functional analysis. B) Western blot analysis showing *ITGA8* protein levels in brain microvessels of *ITGA8*iPCKO and *ITGA8^fl/fl^* mice at 12 days post‐tamoxifen administration. M indicates marker (*n* = 4 mice, *p* = 0.0001). C) Kaplan–Meier survival curve comparing survival rates between *ITGA8*iPCKO and *ITGA8^fl/fl^* mice (> 95%, 60 days). Survival percentages are indicated (*n* = 20 mice, *p* < 0.0001). Log‐rank test. D–F) Gait analysis using the Catwalk XT10.0 system. Quantification of regularity index D), maximum contact area E), and swing speed F) in *ITGA8*iPCKO and *ITGA8^fl/fl^* mice at 40 days. Right forelimb (RF), right hindlimb (RH), left forelimb (LF), and left hindlimb (LH) are assessed (*n* = 20 mice, *p* = < 0.0168, 0.0001, 0.0012, 0.0002, 0.0148, 0.0008, 0.0001, 0.0001, and 0.0001). G) Rotarod test results showing motor coordination and learning capabilities, with latency to fall for *ITGA8*iPCKO and *ITGA8^fl/fl^* mice at 12‐ and 40‐days (*n* = 20 mice, *p* = 0.0023). H,I) Pole Climbing Test (PCT) results measuring turnaround time (iTurn) H) and total climbing duration (TD) I) at 12‐ and 40‐days (*n* = 18 mice, all *p* < 0.0001). J) Timeline of Morris Water Maze (MWM) test was conducted on *ITGA8*iPCKO mice and *ITGA8^fl/fl^* mice. K) Initial swimming distances was measured in the MWM test before training trials at 40 days. Data from *ITGA8^fl/fl^* (*n* = 13 mice) and *ITGA8*iPCKO (*n* = 27 mice, *p* = 0.5132). L) Comparison of escape latency during the acquisition phase of the MWM test between *ITGA8*iPCKO and control mice at different time points. Data from *ITGA8^fl/fl^* (*n* = 13 mice) and *ITGA8*iPCKO (*n* = 27 mice, *p* = 0.6962, 0.0212, 0.0873, 0.0042, 0.013). M) Representative swimming traces of *ITGA8*iPCKO and *ITGA8^fl/fl^* mice during the probe trial at 45 days. N,O) Quantification of platform zone crossings N) and latency to first reach the platform zone O) during the probe trial at 45 days in the MWM test. Data from *ITGA8^fl/fl^* (*n* = 13 mice) and *ITGA8*iPCKO (*n* = 27 mice, *p* = 0.0388, 0.0332). P) Golgi staining of pyramidal neurons in the motor cortex and hippocampus CA1 at 40 days. Scale bar: 50 µm. Higher magnification of dendritic spines shown in right panels. Scale bar: 15 µm. Q) Quantitative analysis of dendritic spine density in neurons of the motor cortex and hippocampus CA1 at 40 days. Data points represent mice. *ITGA8*iPCKO (*n* = 8 mice), *ITGA8^fl/fl^* (*n* = 5 mice, all *p* < 0.0001). R) Fluoro‐Jade B (FJB) staining of brain sections at 40 days. FJB positive neurons are indicated by white arrowheads. (Scale bar: 30 µm, *n* = 6 mice). Data represent mean ± SEM. Significance notations: ns (*p* > 0.05), ^*^ *p* < 0.05; ^**^ *p* < 0.01; ^***^ *p* < 0.001; ^****^ *p* < 0.0001. All intergroup comparisons were analyzed using unpaired 2‐tailed Student *t‐*test.

To delineate *ITGA8*’s role in pericyte‐mediated BBB maintenance, we developed an inducible pericyte‐specific *ITGA8* knockout model (*Pdgfrb*‐ERT‐Cre; *ITGA8^fl/fl^*, termed *ITGA8*iPCKO) through Cre‐loxP recombination (Figure S3A,B, Supporting Information). Following tamoxifen‐induced Cre activation initiated at 7–8 weeks of age, *ITGA8* was selectively ablated in PDGFRβ+ cells, as confirmed by Western blot (**Figure** **2**A,B). In our observation of *ITGA8*iPCKO mice, a progressive decline in their overall health, mobility, and physiological state became evident (Videos S1–S4), culminating in significantly reduced survival compared to controls within two months (Figure 2C). To encompass both early and intermediate‐to‐late responses post‐*ITGA8* gene deletion, we established 12‐day and 40‐day as critical time points for functional analyses (Figure 2A).

Comprehensive behavioral phenotyping revealed progressive neurological dysfunction in *ITGA8*iPCKO mice. CatWalk gait analysis demonstrated significant deterioration in locomotor coordination by day 40, manifesting as reduced regularity index, diminished maximum contact area, and impaired swing speed (Figure 2D–F). Motor learning deficits emerged earlier, with rotarod testing showing decreased fall latency at both 12‐ and 40‐day timepoints (Figure 2G). Pole climbing tests (PCT) confirmed progressive motor impairment through increased turnaround time (iTurn) and total duration (TD) (Figure 2H,I). Spatial memory assessment via Morris water maze revealed preserved baseline swimming ability (Figure 2J,K; Figure S3C, Supporting Information) but impaired acquisition learning (Figure 2L; Figure S3D, Supporting Information). Probe trials revealed memory recall deficits in *ITGA8*iPCKO mice, characterized by fewer platform zone crossings and extended times to locate the platform at 45 days (Figure 2M–O). Notably, even at day 17 (an earlier time point), mutants exhibited fewer platform zone crossings despite similar probe times, suggesting early cognitive deficits (Figure S3E–G, Supporting Information).

Neuropathological analysis supported these behavioral findings. Golgi staining demonstrated significant dendritic spine loss in motor cortex pyramidal neurons and hippocampal CA1 granule cells (Figure 2P,Q; Figure S3H,I, Supporting Information) at both 12‐ and 40‐days, with pan‐cerebral distribution observed in whole‐brain analyses (Data not shown). Correspondingly, synaptic marker proteins Homer1 and Bassoon were reduced in these regions (Figure S3K–N, Supporting Information), indicating synapse loss. Fluoro‐Jade B (FJB) staining, which labels degenerating neurons, was negative in mutants at 12 days but revealed pronounced neuronal degeneration in motor cortex and CA1 by 40 days (Figures 2R, S3J). These degenerative changes extended to basal ganglia and cortical regions (Data not shown). Taken together, these results demonstrate that pericyte *ITGA8* is crucial for maintaining normal neural function, as its loss leads to widespread neurodegeneration, and behavioral deficits.

### 3.3 Pericytes *ITGA8* Controls BBB Integrity

**Figure 3:**
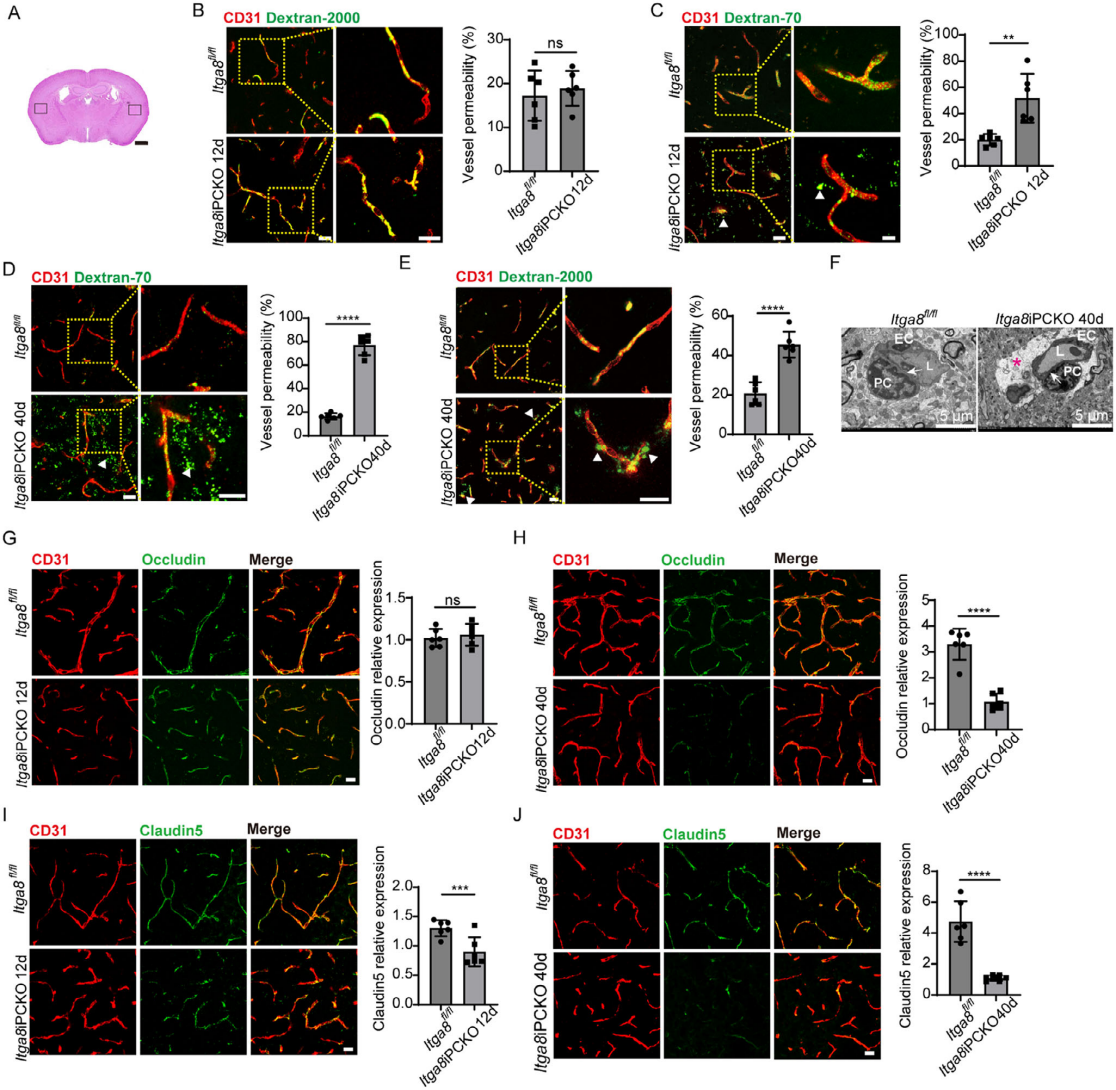
Role of Pericyte Itga8 in Maintaining BBB Integrity. A) Hematoxylin and eosin–stained coronal sections. The framed areas show the location used for all histological measurements. Scale bar: 1000 µm. B) The brain vessels in *ITGA8*iPCKO mice and *ITGA8^fl/fl^* mice show the comparable permeability to dextran‐2000 kDa at 12 days. Higher magnification views in right panels. Scale bar: 25 µm. Quantification of vessel permeability to dextran‐2000 kDa (*n* = 6 mice, *p* = 0.5768). C) Brain sections of *ITGA8*iPCKO and *ITGA8^fl/fl^* mice at 12 days showing dextran‐70 kDa leakage from blood vessels. Higher magnification views in right panels. Scale bars: 25 µm (left), 10 µm (right). Arrowheads point to dextran‐70 kDa tracer leakage outside blood vessels. Vessel permeability was quantified in brain sections (*n* = 6 mice, *p* = 0.0021). D) Brain sections of *ITGA8*iPCKO and *ITGA8^fl/fl^* mice at 40 days showing dextran‐70 kDa leakage from blood vessels. Higher magnification views in right panels. Scale bar: 25 µm (left). Arrowheads point to dextran‐70 kDa tracer leakage outside blood vessels. Vessel permeability was quantified in brain sections (*n* = 6 mice, *p* < 0.0001). E) Brain sections of *ITGA8*iPCKO and *ITGA8^fl/fl^* mice at 40 days showing leakage of dextran‐2000 kDa from blood vessels. Higher magnification in right panels; Arrowheads point to dextran‐2000KD tracer leakage outside blood vessels. Scale bar: 25 µm. Quantification of vessel permeability in brain sections (*n* = 6 mice, *p* < 0.0001). F) Transmission electron microscopy of brain capillaries in *ITGA8*iPCKO and control mice at 40 days. EC: endothelial cell; PC: pericyte; L: lumen. Arrows indicate the basement membrane; the red asterisk (*) highlights perivascular edema. G,H) Double immunostaining of CD31 and Occludin in brain sections at 12 days G) and 40 days H). Scale bar: 20 µm. Right panels: Quantification of Occludin mean fluorescence intensity (MFI) on CD31+ vessels (*n* = 6 mice, *p* = < 0.5932, 0.0001). I,J) Double immunostaining of CD31 and Claudin5 in brain sections at 12 days I) and 40 days J). Scale bar: 20 µm. Right panels: Quantification of Claudin5 MFI on CD31+ vessels (*n* = 6 mice, *p* = < 0.0062, 0.0001). Data represent mean ± SEM. Significance notations: ns (*p* > 0.05); ^**^ *p* < 0.01; ^***^ *p* < 0.001; ^****^ *p* < 0.0001. All intergroup comparisons were analyzed using unpaired 2‐tailed Student *t‐*test.

Given the observed neural deficits in *ITGA8*iPCKO mice, we next investigated BBB integrity in these mutants following tamoxifen‐induced knockout at 7–8 weeks of age. We performed in vivo tracer extravasation assays using high molecular weight (2000 kDa) and low molecular weight (70 kDa) fluorescent dextran at 12‐ and 40‐days. As shown in **Figure** **3**A, the framed area was the region where we exhibited the microvessels phenotypes. While 2000 kDa dextran leakage remained comparable between genotypes at 12 days (Figure 3B), *ITGA8*iPCKO mice exhibited significantly increased 70 kDa tracer penetration, indicating early‐stage BBB compromise (Figure 3C). By day 40, mutant mice demonstrated progressive deterioration with exacerbated 70 kDa leakage (vs day 12) and de novo 2000 kDa extravasation (Figure 3D,E), phenotypes showing pan‐cerebral distribution (Data not shown). Ultrastructural examination by electron microscopy showed evidence of BBB breakdown, such as perivascular edema surrounding capillaries in mutants (Figure 3F). Immunostaining for tight junction proteins revealed molecular correlates of barrier dysfunction. In *ITGA8*IPCKO mice, endothelial junctional proteins claudin‐5 and occludin were progressively downregulated at 40 days, whereas zonula occludens‐1 (ZO‐1) levels remained unchanged (Figure 3G,J; Figure S4A,B, Supporting Information). These results underscore the essential role of ITGA8 in sustaining BBB integrity. Interestingly, the vascular phenotype appeared brain‐specific: we assessed microvascular permeability in peripheral organs (kidney, heart, and lungs) using 70 kDa and 2000 kDa dextrans and found no significant differences between *ITGA8*iPCKO and wild‐type mice (Figure S4C–E, Supporting Information). This suggests either that *ITGA8* loss does not critically impact peripheral microvascular barriers, or that these organs require even larger tracers or more sensitive assays to detect subtle changes. In either case, the BBB of the central nervous system is uniquely vulnerable to pericyte *ITGA8* deletion, indicating a potentially brain‐specific requirement for *ITGA8* in microvascular stability.

### 3.4 *ITGA8* Regulates the Morphology of Pericytes on the Microvessels

**Figure 4:**
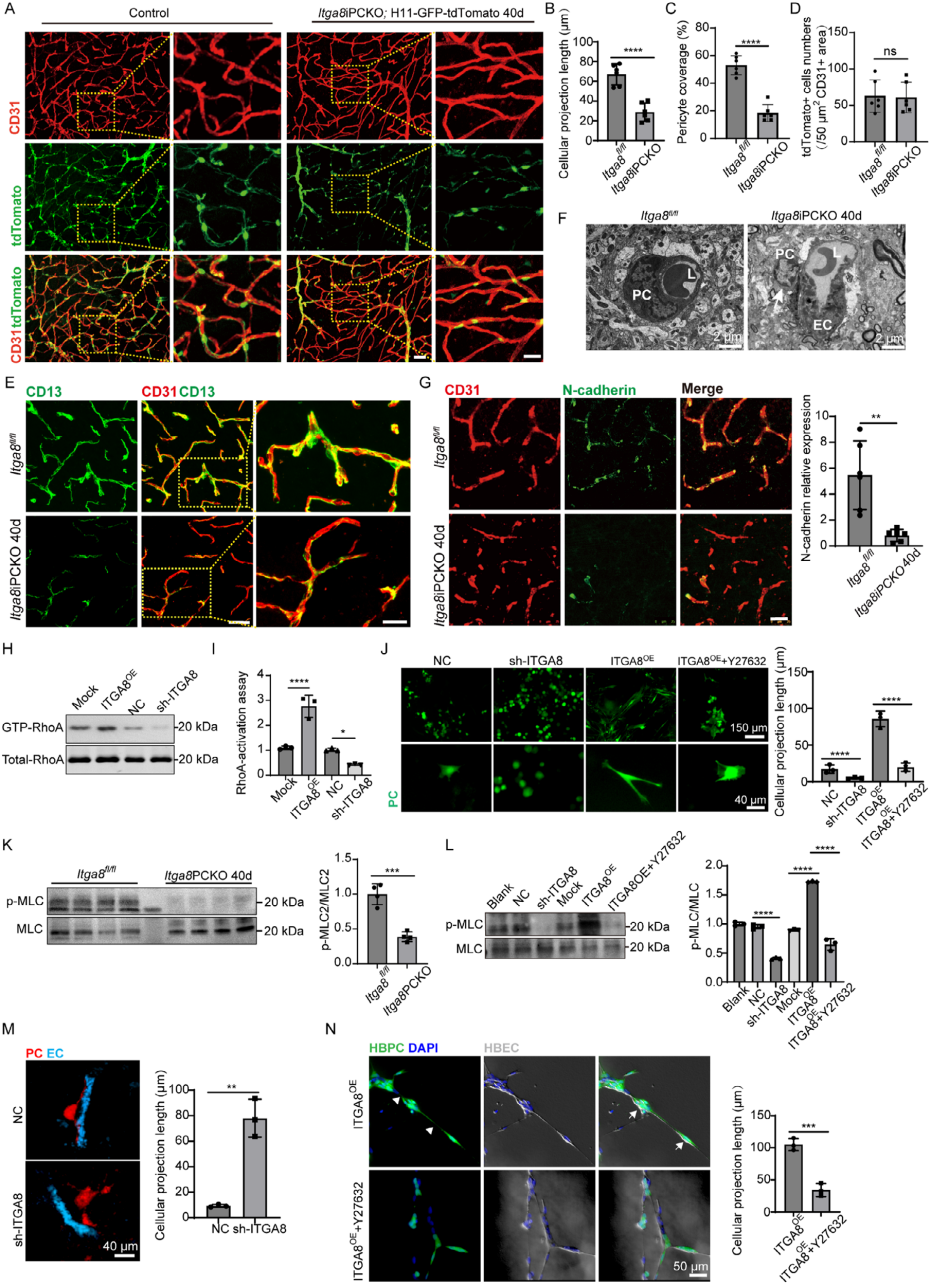
Impact of *ITGA8* Deficiency on Pericyte Morphology and Interactions with Endothelial Cells. A) Brain sections of *ITGA8*iPCKO; H11‐GFP‐tdTomato mice and control mice at 40 days, immunostained for vessels. Scale bar: 40 µm. Higher magnification images in the right panels indicated by yellow boxes, Scale bar: 20 µm. B–D) Quantification of B) pericyte projection length, C) coverage, and D) number in brain sections at 40 days (*n* = 6 mice, *p* = < 0.0001, 0.0001, 0.8753). E) Representative images of CD13+ pericyte coverage on CD31+ vessels in brain sections of *ITGA8*iPCKO mice and control mice at 40 days. Scale bar: 40 µm. Higher magnification in right panels with yellow boxes, Scale bar: 20 µm. F) Transmission electron microscopy of brain microvessels in *ITGA8*iPCKO and *ITGA8^fl/fl^* mice at 40 days. EC: endothelial cell; PC: pericyte; L: lumen. The arrowhead indicates contracted pericyte bodies and diminished pericyte coverage on microvasculature, with pericytes appearing reduced interactions with neighboring endothelial cells in *ITGA8*iPCKO mice. G) Double immunostaining for CD31 and N‐cadherin in brain sections at 40 days. Scale bar: 25 µm. Quantitative analysis of N‐cadherin relative MFI on CD31+ vessels in brain sections at 40 days (*n* = 6 mice, *p* = 0.0018). H) GTP‐loaded RhoA levels measurement using a RhoA activation assay kit, which employs the pull‐down method to quantify active RhoA levels in HBPCs following hITGA8 and its knockdown (*n* = 3 independent experiments). I) Quantification of RhoA activation status in HBPCs under *ITGA8* overexpression versus knockdown conditions was performed using the G‐LISA RhoA activation assay (*n* = 3 independent experiments, *p* = <0.0001, 0.0470). J) Images of ITGA8 manipulated HBPCs show that *ITGA8* knockdown in HBPCs results in altered cell shapes and reduced projections compared to control cells, while *ITGA8* overexpression enhanced cellular extensions. Additionally, the application of the ROCK inhibitor Y27632 to *ITGA8*‐overexpressing HBPCs reveals a reduction in the *ITGA8*‐induced morphological changes (*n* = 3 independent experiments, all *p* < 0.0001). K) Western blot analysis of p‐MLC/MLC levels in the pericytes of *ITGA8*iPCKO and *ITGA8^fl/fl^* mice at 40 days (*n* = 4 mice, *p* = 0.0003). L) Western Blot Analysis of p‐MLC in HBPCs depicting the levels of p‐MLC/MLC in HBPCs following *ITGA8* knockdown or overexpression or treated with RhoA inhibitor Y27632 (*n* = 3 independent experiments, all *p* < 0.0001). M) Confocal images of HBPC‐HBEC mixed cultures in fibrin gel on day 3, showing cellular interactions. HBPCs were labeled with a red CellTracker dye and HBECs with a green one (*n* = 3 independent experiments, *p* = 0.0012). N) Confocal image showing interactions between GFP‐expressing HBPCs (green) and unlabeled HBECs following a 16 h co‐culture in Matrigel. Arrowheads point to altered cellular projections in pericytes. Arrowheads denote pericyte processes and intercellular networks, while arrows highlight endothelial‐aligned pericyte somata (*n* = 3 independent experiments, *p* = 0.0009). Data represent mean ± SEM. Significance notations: ns (*p* > 0.05), ^**^ *p* < 0.01, ^****^ *p* < 0.0001; Unpaired, 2‐tailed Student *t‐*test was used to compare groups in (B–D), (G), (K), and (N). Comparisons between multiple groups were made using one‐way ANOVA test followed by Tukey's HSD post hoc test in (I–J) and (L).

To investigate the role of *ITGA8* in PDGFRβ+ mural cells during BBB disruption, we generated *Pdgfrb*‐ERT‐Cre; *ITGA8^fl/fl^*; H11‐tdTomato‐GFP (*Itga8*iPCKO; *Pdgfrb*‐tdTomato) mice, enabling in vivo tracking and analysis of *ITGA8*‐deficient PDGFRβ+ cells through fluorescent labeling (Figure S3A, Supporting Information). Notably, no significant alterations in the shape or coverage of smooth muscle cells (SMCs) on arterioles were detected in *ITGA8*iPCKO mice at either 12‐ or 40‐day post‐induction (Figure S5A, Supporting Information). No changes in the shape, number, or coverage of pericytes on microvasculature were evident in mutant mice at 12 days (Figure S5B–D, Supporting Information). However, by 40 days, notable changes in pericyte processes emerged, leading to diminished coverage on microvasculature and disruption in the pericyte network in *ITGA8*iPCKO mice (**Figure** **4**A,C). These *ITGA8*‐deficient pericytes exhibited shorter process, irregular distribution patterns, and lacked proper alignment along the basement membrane (Figure 4A,B). Despite these morphological changes, pericyte number and microvascular area remained unchanged at 40 days (Figure 4D; Figure S5E,F, Supporting Information). CD13 immunostaining corroborated the alterations in pericyte processes and reduced vessel coverage in *ITGA8*iPCKO mice at this time point (Figure 4E). Electron transmission analysis further confirmed these findings, showing contracted pericyte bodies, diminished pericyte coverage, and loosely attached pericytes on the microvascular surface (Figure 4F). N‐cadherin immunostaining showed markedly decreased pericyte‐endothelial junctions in 40‐day *ITGA8*iPCKO mice vs controls (Figure 4G). Basement membrane analysis revealed a time‐dependent decline in collagen 4 and laminin expression in *Itga8*iPCKO mice, showing comparable levels to controls at 12 days but marked decreases by 40 days (Figure S5G–J, Supporting Information). Whole‐brain analyses revealed simultaneous pericyte morphological alterations, reduced microvessel coverage, and decreased laminin/collagen4 deposition (data not shown). These results emphasize the essential role of ITGA8 in maintaining pericyte normal morphology and association between endothelial cells and pericytes.

Having observed that deletion of ITGA8 resulted in shorter pericyte processes, we sought to uncover the underlying mechanism. RhoA–ROCK signaling is a master regulator of actomyosin dynamics,^[^ ^44^, ^45^ ^]^ we hypothesized that ITGA8 might influence the pericyte cytoskeleton through this pathway. Lentiviral manipulation of ITGA8 expression (Figure S5K, Supporting Information) revealed its bidirectional regulation of RhoA activation: overexpression increased GTP‐RhoA levels, while knockdown reduced active RhoA compared to controls (Figure 4H). G‐LISA RhoA activation assays further corroborated these results (Figure 4I). The data position ITGA8 upstream of RhoA in regulating cytoskeletal dynamics, explaining the observed defects in pericyte adhesion and morphogenesis.

We next examined morphological outcomes of RhoA modulation in pericytes. In GFP‐labeled primary pericytes, *ITGA8* knockdown induced a rounded cell shape with loss of processes, whereas *ITGA8* overexpression promoted an elongated, arborized morphology (Figure 4J). Treating *ITGA8*‐overexpressing cells with the ROCK inhibitor Y27632 reversed the elongated phenotype, causing cells to resemble controls (Figure 4J). This confirms that RhoA–ROCK signaling is a key downstream effector of *ITGA8* in shaping the pericyte cytoskeleton. In vivo, phosphorylation of myosin light chain (p‐MLC), a readout of actomyosin contractile tension, was significantly reduced in pericytes from *ITGA8*iPCKO mice compared to controls (Figure 4K), indicating diminished cytoskeletal tension when *ITGA8* is absent. Similarly, in cultured HBPCs, ITGA8 knockdown lowered p‐MLC levels, while *ITGA8* overexpression increased p‐MLC, and co‐treatment with Y27632 abolished the ITGA8‐induced p‐MLC elevation (Figure 4L).

Subsequently, we established co‐culture systems to study the interactions between HBPCs and human brain endothelial cells (HBECs). We first assessed pericyte‐endothelial interactions using a fibrin gel co‐culture system containing HBPCs (with or without *ITGA8* knockdown) and HBECs. Control HBPCs exhibited characteristic elongated morphology with extensive projections that closely aligned with endothelial networks, whereas *ITGA8*‐knockdown HBPCs demonstrated markedly reduced process extension and diminished physical contact with HBECs (Figure 4M), indicating compromised intercellular communication. To further investigate the mechanistic basis, we employed a Matrigel co‐culture system with *ITGA8*‐overexpressing HBPCs. These cells formed well‐organized networks with endothelial structures through enhanced cellular projections. Strikingly, treatment with the ROCK inhibitor Y27632 reversed this phenotype–*ITGA8*‐overexpressing pericytes displayed blunted processes and disrupted alignment with endothelial cells (Figure 4N). This pharmacological intervention conclusively demonstrated that *ITGA8* mediates pericyte‐endothelial interaction through Rho‐ROCK‐dependent cytoskeletal remodeling.

### 3.5 ITGA8‐Dependent Cytoskeletal Tension Triggers Latent TGF‐β1 Activation

**Figure 5:**
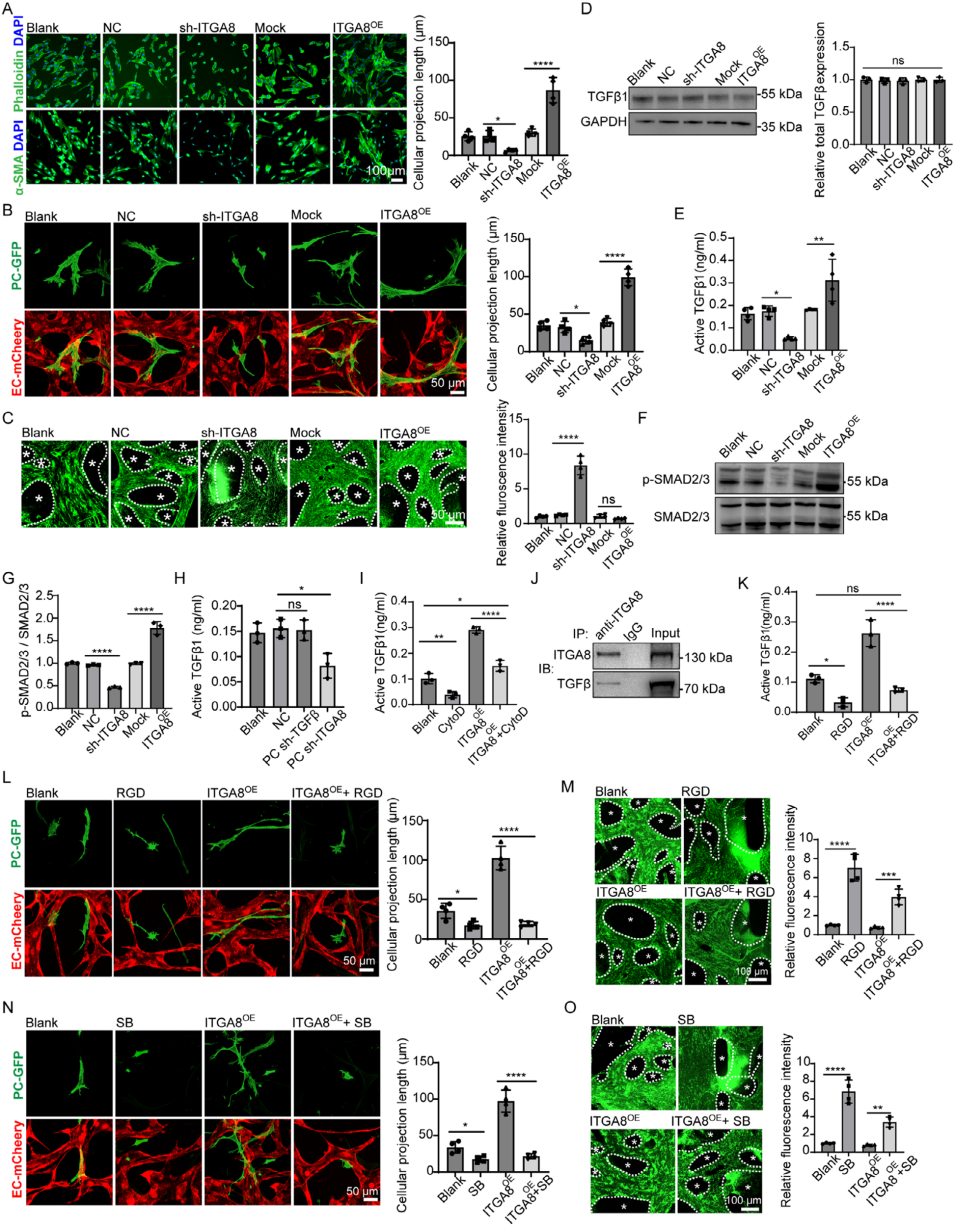
Pericyte Cytoskeletal Tension and Interaction with ECM Activating TGFβ Signaling. A) Immunofluorescence analysis of F‐actin (phalloidin) and α‐SMA in pericytes demonstrated *ITGA8* expression‐dependent cytoskeletal remodeling, with quantitative assessment of phalloidin‐stained cellular projections (*n* = 4 independent experiments, *p* = < 0.0421, 0.0001). B) Representative images of the vascular network formed by HBECs (lenti‐mCherry‐labeled) and HBPCs (lenti‐EGFP‐labeled) in microfluidic chips are shown. HBPCs with ITGA8 knockdown or overexpression exhibited morphological changes and altered interaction with endothelial cells (*n* = 4 independent experiments, *p* = < 0.0286, 0.0001). C) Representative fluorescent images showing the leaked FITC‐microbeads outside of vascular at 60 min post‐perfusion. The asterisks indicate the area outside of the vascular (*n* = 4 independent experiments, *p* = < 0.0001, 0.9325). D) Western blotting analysis showing the total *TGFβ1* levels after *ITGA8* knockdown or overexpression in HBPCs. The total *TGFβ1* levels were quantified in different group (*n* = 3 independent experiments, *p* = 0.9686). E) Elisa analysis showing the active TGFβ in the HBPCs conditional medium following *ITGA8* knockdown or overexpression (*n* = 3 independent experiments, *p* = 0.0123, 0.0066). F,G) Western blot analysis of p‐SMAD2/3 / SMAD2/3 levels in HBPCs following *ITGA8* knockdown or overexpression (*n* = 3 independent experiments, all *p* < 0.0001). H) Elisa analysis of active *TGFβ1* levels in the condition medium of pericyte‐endothelial cell transwell co‐culture system (*n* = 3 independent experiments, *p* = 0.9971, 0.0102). I) Elisa analysis showing the active TGFβ levels in the HBPCs conditional medium following *ITGA8* overexpression or treatment with CytoD (*n* = 3 independent experiments, *p* = < 0.0083, 0.0276, 0.0001). J) Co‐immunoprecipitation (Co‐IP) showing the binding between *ITGA8* and *TGFβ1*. K) Elisa analysis showing the mature TGFβ in the HBPCs conditional medium after pretreatment with RGD peptide (*n* = 3 independent experiments, *p* = < 0.0194, 0.3252, 0.0001). L) Representative images of the vascular network formed by HBECs (lenti‐mCherry‐labeled) and HBPCs (lenti‐EGFP‐labeled) in microfluidic chips are shown. HBPCs were transfected with lenti‐*ITGA8* or the co‐culture system were treated with RGD peptide (*n* = 4 independent experiments, *p* = < 0.0446, 0.0001). M) Representative fluorescent images showing the leaked FITC‐microbeads outside of vascular at 60 min post‐perfusion in Blank, RGD, *ITGA8* ^OE^, and *ITGA8* ^OE^ + RGD group. The asterisks indicate the area outside of the vascular. The vascular permeability was quantified (*n* = 4 independent experiments, *p* = < 0.0001, 0.0006). N) Representative images of the vascular network formed by HBECs (lenti‐mCherry‐labeled) and HBPCs (lenti‐EGFP‐labeled) in microfluidic chips of Blank, SB431542, *ITGA8* ^OE^, and *ITGA8* ^OE^ + SB431542 group. HBPCs were transfected with lenti‐*ITGA8* or the co‐culture system were treated with SB431542 (*n* = 4 independent experiments, *p* = < 0.0373, 0.0001). O) Representative fluorescent images showing the leaked FITC‐microbeads outside of vascular at 60 min post‐perfusion in Blank, SB431542, *ITGA8* ^OE^, and *ITGA8* ^OE^ + SB431542 group. The asterisks indicate the area outside of the vascular. The vascular permeability was quantified (*n* = 4 independent experiments, *p* = < 0.0001, 0.0012). Data represent mean ± SEM. Significance notations: ns (*p* > 0.05), ^*^ *p* < 0.05, ^**^ *p* < 0.01, ^****^ *p* < 0.0001; Multi‐group comparisons were analyzed by one‐way ANOVA with Tukey's HSD post‐hoc testing.

To further demonstrate how ITGA8 influences microvascular function, we performed a series of in vitro experiments. *ITGA8* knockdown in pericytes disrupted actin organization and cell morphology, while overexpression enhanced actin fiber formation and elongation (**Figure** **5**A). Functional assays showed ITGA8 knockdown increased migration and reduced adhesion– phenotypes reversed by ITGA8 overexpression (Figure S6A–C, Supporting Information). In co‐culture systems, *ITGA8*‐overexpressing pericytes developed extended projections and strengthened endothelial interactions, contrasting with the diminished protrusions and impaired cell‐cell contacts in knockdown models (Figure 5B). Furthermore, *ITGA8* knockdown elevated vascular permeability to FITC‐microbeads (GF100C, Huge biotechnology), whereas overexpression did not show evident improved barrier integrity (Figure 5C). *ITGA8* predominantly forms functional heterodimers with integrin β1 (*ITGB1*) to mediate cellular interactions.^[^ ^46^, ^47^ ^]^ To delineate the functional interplay between these subunits, we employed lentivirus‐mediated bidirectional modulation (knockdown and overexpression) of *ITGB1* in pericytes (Figure S6D, Supporting Information). Notably, both genetic manipulations recapitulated the morphological alterations previously observed with *ITGA8* modulation (Figure S6E,F, Supporting Information). Furthermore, using a transwell co‐culture system, we showed that knockdown of *ITGB1* markedly elevated endothelial barrier permeability, whereas its overexpression did not significantly enhance barrier integrity (Figure S6G, Supporting Information). Together, these results indicate a functional codependency between *ITGA8* and *ITGB1*.

TGF‐β1 critically regulates vascular homeostasis and BBB integrity through mechanotransduction pathways, where cytoskeletal tension and ECM remodeling control its activation.^[^ ^29^, ^48^, ^49^ ^]^ Given that *ITGA8* regulates pericyte cytoskeleton through the ROCK signaling pathway, it is likely that *ITGA8* influences TGF‐β activation by modulating these mechanical stresses and ECM interactions. In cultured HBPCs, neither *ITGA8* knockdown nor overexpression altered total TGF‐β1 levels (Figure 5D). However, ELISA revealed decreased active TGF‐β1 in *ITGA8*‐deficient HBPCs’ conditioned medium and increased levels in *ITGA8*‐overexpressing HBPCs cultures (Figure 5E). Consistent results showed that *ITGA8* knockdown reduced SMAD2/3 phosphorylation while its overexpression enhanced this response (Figure 5F,G), demonstrating that *ITGA8* regulates TGF‐β1 signaling through activation of the latent complex. Notably, this regulatory effect appeared specific to the canonical TGF‐β/SMAD pathway, as systematic evaluation of non‐canonical TGF‐β1—associated pathways—including MAPK, PI3K‐Akt, and JAK‐STAT signaling cascades—revealed no detectable changes following either genetic suppression or overexpression of ITGA8 (Figure S6H—J, Supporting Information). Using transwell co‐cultures with endothelial cells, we found pericyte *ITGA8* knockdown reduced activated TGF‐β1 levels despite endothelial TGFβ1 expression, while pericyte‐specific TGFβ1 knockdown showed no effect (Figure 5H). This demonstrates pericyte ITGA8 can activate both autocrine and paracrine latent TGFβ1 pools from neighboring endothelial cells.

To elucidate the role of the cytoskeleton in mediating TGF‐β1 activation, pericytes were treated with the cytoskeletal inhibitor Cytochalasin D (Cyto D).^[^ ^49^ ^]^ Our data revealed that cytoskeletal inhibition significantly reduced active TGF‐β1 levels in both *ITGA8*‐overexpressing and control pericytes under mechanical stretch (Figure 5I). Notably, *ITGA8*‐overexpressing cells treated with Cyto D maintained higher active TGF‐β1 compared to blank control, suggesting additional cytoskeleton‐independent activation mechanisms mediated by *ITGA8*. Co‐immunoprecipitation showed the interaction between *ITGA8* and TGFβ1 (Figure 5J). To investigate this interaction mediated TGFβ1 activation, we employed RGD peptide to block *ITGA8*–TGFβ1 interactions. This intervention caused a substantial reduction in the levels of active TGF‐β1 in the conditioned medium from both *ITGA8*‐overexpressing and control pericytes (Figure 5K). However, in contrast to Cyto D, RGD peptide treated *ITGA8*‐overexpressing cells showed lower levels of active TGFβ1 compare to the blank control (Figure 5K). These results suggest *ITGA8* coordinates both cytoskeletal tension‐dependent and matrix interaction‐mediated pathways for latent TGF‐β1 activation.

*ITGA8*‐overexpressing HBPCs displayed elongated morphologies with extended cellular projections, which were disrupted by RGD peptide treatment through actin fiber disorganization (Figure S7A, Supporting Information). The RGD peptide enhanced pericyte migration and reduced adhesion, mimicking *ITGA8* knockdown effects (Figure S7B–D, Supporting Information). Notably, these phenotypic changes persisted even in *ITGA8*‐overexpressing cells (Figure S7B–D, Supporting Information). In co‐culture systems, RGD‐treated pericytes exhibited diminished projections and endothelial association, irrespective of *ITGA8* overexpression status (Figure 5L). Furthermore, RGD exposure increased vascular permeability to FITC‐microbeads, counteracting the vascular stabilization conferred by *ITGA8* overexpression (Figure 5M).

To further corroborate the important role of TGFβ1 signaling pathway in regulating BBB function, we applied a TGFβ signaling pathway inhibitor SB431542. SB431542 significantly altered the morphology of pericytes, enhanced its migration ability, and inhibited the adhesion ability, irrespective of *ITGA8* overexpression (Figure S7E–H, Supporting Information). In vitro co‐culture system showed that SB431542 resulted in reduced prostrations of pericytes and compromised physical interaction between pericytes and endothelial cells (Figure 5N). In addition, we observed increased permeability of the vascular in vitro in the SB431542 treated *ITGA8*‐overexpressing group (Figure 5O). These findings collectively show that *ITGA8* plays a pivotal role in regulating pericyte function and vascular function through activating latent TGFβ1 by cytoskeletal tension and ECM interactions.

### 3.6 Dynamic Expression Pattern of *ITGA8* Post‐Stroke

**Figure 6:**
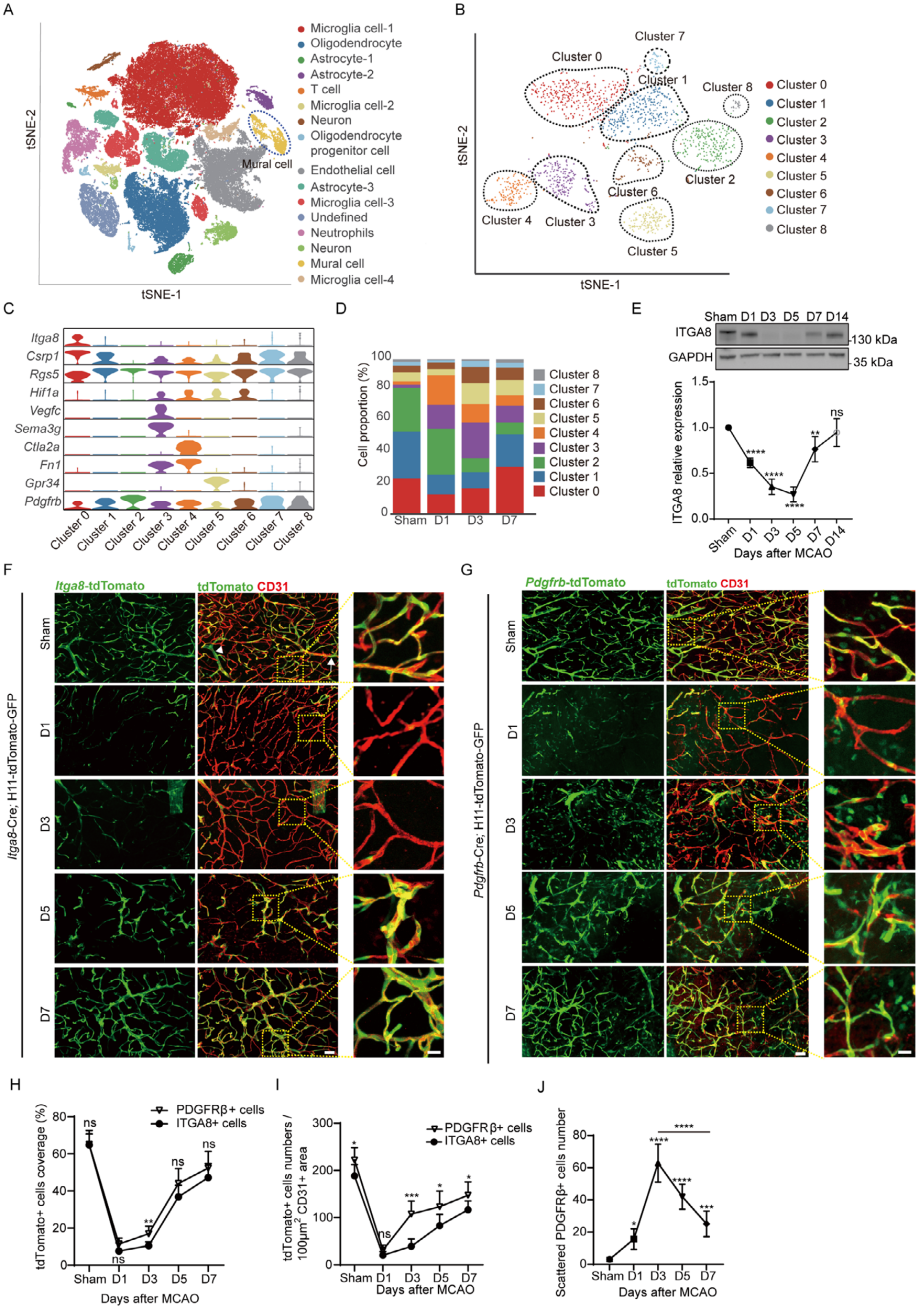
Temporal Dynamics of ITGA8 Expression and Localization Post‐Stroke. A) tSNE plots of total 71040 cells derived from MCAO model and sham‐operated controls, revealing 16 major clusters from brain tissues collected at days 1, 3, and 7 post‐MCAO, as well as from the sham group with three mice included in each group. Each point in the plot represents an individual cell, indicated by a droplet barcode and assigned a color based on its cluster. 1804 mural cells were identified. B) tSNE‐based unsupervised clustering revealing nine unique mural cell subtypes, each differentiated by distinct gene expression profiles. C) Violin plots show the expression of top cell‐type specific marker genes in each cluster of mural cells. D) Dynamic changes in mural cell subtype proportions post‐ischemia. Graph showing the temporal variation of specific mural cell subtypes (including cluster 0) at different time points (Sham, Day 1, 3, and 7 following MCAO surgery). E) Western blotting analysis of ITGA8 expression post‐stroke (*n* = 6 mice, *p* = < 0.0001, 0.0001, 0.0001, 0.0012, 0.8101). F) Immunofluorescence staining demonstrates the distinct temporal pattern of ITGA8+ cell in post stroke. Scale bar: 40 µm. Right panels are magnified views of boxes indicated in left panels. Scale bar: 15 µm. Arrowhead indicates arteriole, arrow indicates venule. G) Immunofluorescence staining demonstrates the temporal pattern of PDGFRβ+ cell after stroke. Scale bar: 40 µm. Right panels are magnified views of boxes indicated in left panels. Scale bar: 15 µm. H) Quantification of tdTomato+ cells coverage on vessels of *ITGA8‐*Cre; H11‐tdTomato‐GFP mice and *Pdgfrb*‐Cre; H11‐tdTomato‐GFP mice at the same time timepoint, including Sham, D1, D3, D5 and D7 post‐stroke (*n* = 6 mice, *p* = 0.9841, 0.1232, 0.0056, 0.1523, 0.6488). I) Quantification of tdTomato+ cells per 100 µm^2^ CD31+ area in different brain regions of *ITGA8*‐Cre; H11‐tdTomato‐GFP mice. Comparison of tdTomato+ cell density between two genotypes at the same timepoint (*n* = 6 mice, *p* = 0.0431, 0.0737, 0.0003, 0.0378, 0.0484). J) Quantification of Scattered PDGFRβ+ cells at different time point. The number of tdTomato+ cells was compared across different time points (*n* = 6 mice, *p* = < 0.0342, 0.0001, 0.0001, 0.0002, 0.0001). Data represent mean ± SEM. Significance notations: ns (*p* > 0.05), ^**^ *p* < 0.01, ^***^ *p* < 0.001, ^****^ *p* < 0.0001; Unpaired, 2‐tailed Student *t‐*test was used to compare groups in (H,I). Comparisons between multiple groups were made using one‐way ANOVA test followed by Tukey's HSD post hoc test in (E) and (J).

Brain disorders lead to a notable disruption of the BBB, along with evident changes in pericyte phenotype.^[^ ^5^, ^50^ ^]^ To investigate injury‐induced cellular dynamics, we utilized a middle cerebral artery occlusion (MCAO) mouse model, which mimics diverse brain insults and exhibits BBB restoration during recovery.^[^ ^51^, ^52^ ^]^ Brain tissues from MCAO mice (collected at Day 1, Day 3, Day 7 post‐stroke) and sham controls underwent single‐cell sequencing, yielding 13800 (sham), 12458 (Day 1), 20707 (Day 3), and 24075 (Day 7) cells. After Seurat‐based batch correction and integration, unsupervised clustering with t‐SNE identified 16 transcriptionally distinct cell clusters, classified based on unique gene expression profiles and annotated using well‐established cell‐type markers (**Figure** **6**A; Figure S8A, Supporting Information). Using canonical markers (*Pdgfrb*, *Rgs5*; Figure 6A; Figure S8A, Supporting Information), we isolated vascular mural cells (encompassing smooth muscle cells and pericytes) for subcluster analysis. t‐SNE‐based unsupervised clustering delineated nine transcriptionally distinct mural cell subtypes (Figure 6B), each exhibiting unique functional gene signatures (Figure 6C). Notably, *ITGA8* expression was restricted to Cluster 0 (Figure 6C; Figure S8C, Supporting Information), which demonstrated pronounced temporal dynamics: its abundance markedly decreased at post‐ischemia day 1 (D1) and day 3 (D3), followed by partial recovery by day 7 (D7) (Figure 6D). Conversely, clusters 3–4 peaked at D1–D3, while clusters 5–6 showed transient elevation at D3 (Figure 6D), suggesting subtype‐specific responses to ischemic stress. Moreover, *Hif1a*, associated with hypoxia response, was exclusively expressed in clusters 3–6 (Figure 6C). Notably, its expression was significantly elevated in cluster 3 at D3 post‐ischemia (Figure S8D, Supporting Information). KEGG pathway analysis of the top 50 highly expressed genes in these clusters revealed that cluster 0 was significantly enriched in pathways related to actin cytoskeleton regulation, focal adhesion, and tight junction, consistent with a mature mural cell phenotype (Figure S8E, Supporting Information). Cluster 1 exhibited associations with cGMP‐PKG signaling and vascular smooth muscle contraction; Clusters 3–4 were enriched in HIF‐1α signaling and cell migration pathways, suggesting immature / adaptive phenotypes (Figure S8E, Supporting Information). Collectively, these findings indicate that *ITGA8*‐expressing mural cells (cluster 0), characterized as mature mural cells, and plays a crucial role in promoting BBB integrity in adult mice. Strikingly, *ITGA8* exhibited mural cell‐specific expression within *Pdgfrb*+ populations, distinguishing it from other integrins (*Itga5*, *Itga6*, *Itgav*) (Figure S8B, Supporting Information). Western blotting validated ischemia‐induced *ITGA8* downregulation (lowest at D3–D5) and subsequent recovery at D7 (Figure 6E), aligning with cluster 0 dynamics. In addition, the reduced *ITGA8*’s expression was also observed in human infarct brain tissues (Figure S8F, Supporting Information). Additionally, a consistent reduction in *ITGA8* was observed across other brain pathological conditions in recent single‐cell RNA sequencing datasets.^[^ ^53^, ^54^ ^]^ Collectively, these findings suggest the potential involvement of *ITGA8* in preserving BBB integrity during ischemia.

In light of these findings, probing the dynamic expression, distribution, and morphology of ITGA8‐expressing cells post‐ischemia is essential. In sham‐operated mice, most PDGFRβ+ cells rest on vessel walls, with a few scattered in the brain parenchyma (Figure 6G). We focused our analysis on the peri‐infarct area to track *ITGA8*+ cell dynamics. Post‐stroke, days 1 and 3 see a sharp drop in *ITGA8*‐expressing cells on vessel walls, similar to PDGFRβ+ cells (Figure 6F–I). Meanwhile, dispersed PDGFRβ+ cells in the infarct area surge (Figure 6G,J). By day 5, *ITGA8*‐expressing cells become more prominent, coinciding with more PDGFRβ+ cells on vessels, signaling the start of post‐stroke angiogenesis (Figure 6F–I). At day 7, *ITGA8*‐expressing pericytes on vessel walls increase in number and coverage, paralleling the expansion of PDGFRβ+ cells on vessels and implying the maturation of the newly formed BBB (Figure 6F–I). In contrast, peri‐infarct region's scattered PDGFRβ+ cells decrease by day 7 vs day 3 (Figure 6G,J). Additionally, we isolated PDGFRβ+ brain cells from *ITGA8*‐Cre; H11‐GFP‐tdTomato mice on day 5 post‐stroke using fluorescence‐activated cell sorting and observed similar changing patterns of *ITGA8*+ cells compare to sham‐operated mice (Figure S8G, Supporting Information).

Our single‐cell RNA‐seq data shows that the decrease and subsequent increase in cluster 0 cells and *ITGA8* expression post‐stroke align with the morphological and distribution changes of *ITGA8*+ cells in the brain vasculature. This suggests a potential connection between the molecular features from scRNA‐seq and the in vivo physical and functional changes in mural cells after stroke.

### 3.7 Integrin α8 is Required for Pericyte Remodeling and BBB Formation in Neovascularization

**Figure 7:**
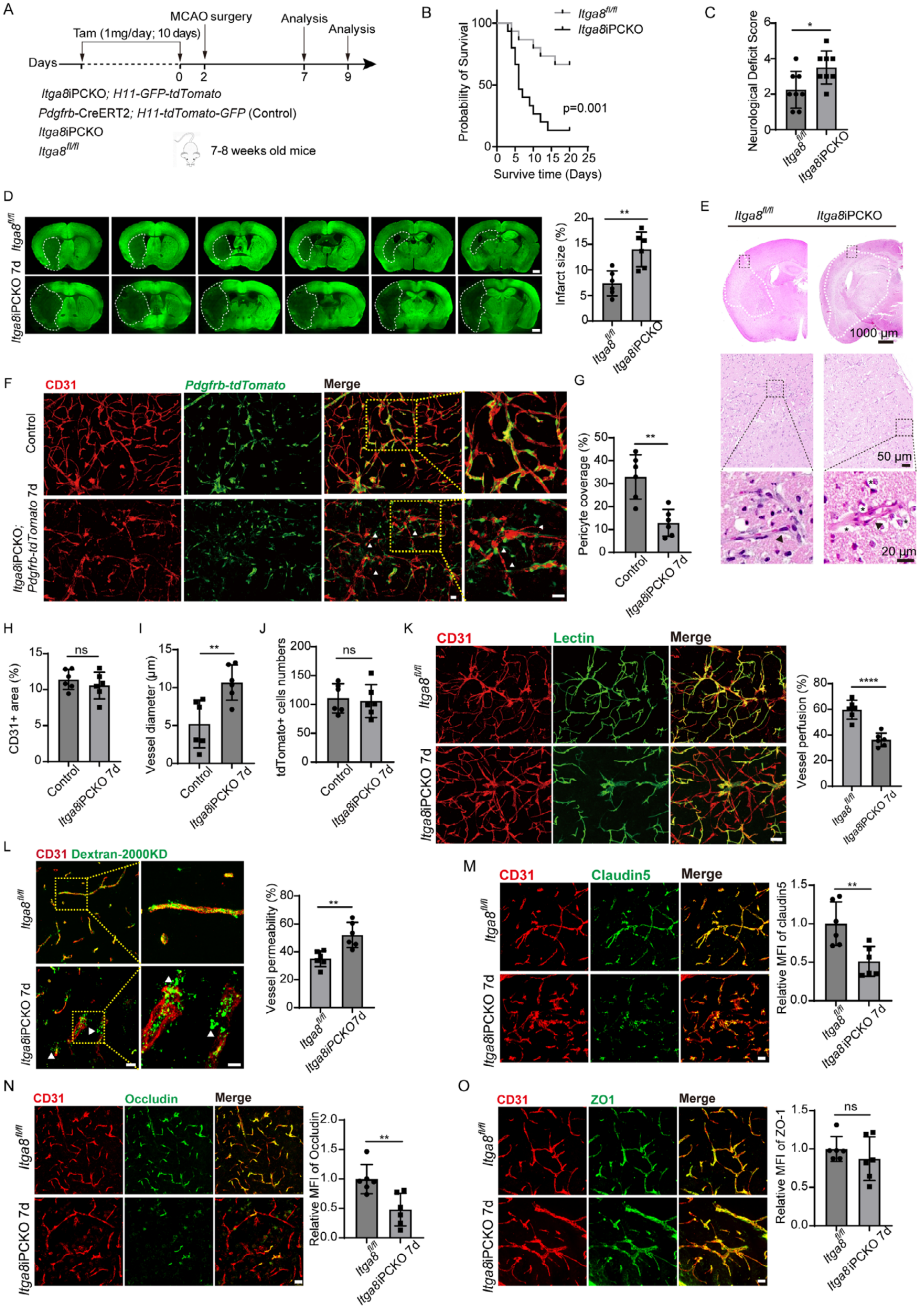
*ITGA8* Regulates Pericyte Remodeling and Neovascularization Post‐Stroke. A) Experimental setup for studying *ITGA8* deletion in pericytes post‐MCAO surgery. B) Kaplan‐Meier survival analysis of *ITGA8*iPCKO mice and control post‐MCAO (> 95%, 20 days). Values are expressed as percentage of surviving mice (*n* = 15 mice, *p* = 0.0010). Log‐rank test. C) Statistical analysis of the neurological deficit score in *ITGA8*iPCKO and *ITGA8^fl/fl^* mice after MCAO surgery at day 7 (*n* = 8 mice, *p* = 0.0233). D) MAP2 immunostaining showing the infarct size of *ITGA8*iPCKO and *ITGA8^fl/fl^* mice, the infarct area was indicated by the yellow broken line. Scale bar: 1000 µm. The infarct size was quantified (*n* = 6 mice, *p* = 0.0030). E) Hematoxylin and eosin–staining showing the abnormal structure of microvascular in *ITGA8*iPCKO mice at day 7. The areas framed by white broken line indicate infarct area. The peri‐infarct area, demarcated by the black frame, was analyzed for vascular phenotypes. The arrowheads indicated the microvascular in the peri‐infarct area. * indicates the perivascular edema. F) Brain sections of *ITGA8*iPCKO; H11‐GFP‐tdTomato mice and control mice at 7 days, immunostained for vessels. Scale bar: 20 µm. The image, with yellow boxes indicating areas of higher magnification in the right panel. Scale bar: 20 µm. Representative image shows abnormalities in pericyte shape and diminished association between pericytes and endothelial cells in *ITGA8*iPCKO mice, highlighted by arrowheads. G–J) Quantification of tdTomato+ pericyte coverage on vessels G), vascular area H), vessel diameter I) and PDGFRβ+ cells number J) at 7 days in *ITGA8*iPCKO mice and control mice (*n* = 6 mice, *p* = 0.0014, 0.4052, 0.0066, 0.7621). K) Lectin perfusion into infarct area vessels was determined. Yellow color (green / red double‐staining) indicates blood perfused vessels. Scale bar: 50 µm. Quantification of vessel perfusion % = Lectin+CD31+ area/total CD31+area*100 (*n* = 6 mice, *p* < 0.0001). L) Dextran‐2000 kDa leaks out of blood vessels in *ITGA8*iPCKO mice subjected to ischemia at 7 days. Scale bar: 20 µm. Yellow boxes correspond to higher magnification images in right panels. Scale bar: 10 µm. Arrowheads show tracer hotspots in *ITGA8*iPCKO mice. Quantification of vessel permeability in brain sections of *ITGA8*iPCKO mice and *ITGA8^fl/fl^* mice (*n* = 6 mice, *p* = 0.0030). M–O) Immunostaining of CD31 and Claudin5 M), Occludin N), and ZO1 O) in the brain sections at 7 days. Scale bar: 25 µm. Tight junctions’ MFI on the microvessels were quantified in *ITGA8*iPCKO and *ITGA8^fl/fl^* mice (*n* = 6 mice, *p* = 0.0062, 0.0058, 0.3632). Data represent mean ± SEM. Significance notations: ns (*p* > 0.05), ^*^ *p* < 0.05; ^**^ *p* < 0.01; ^****^ *p* < 0.0001. Intergroup comparisons were analyzed using unpaired 2‐tailed Student *t‐*test.

Given *ITGA8*’s specific post‐stroke expression and distribution, we explored *ITGA8*’s role in pericyte remodeling and BBB formation during post‐ischemic neovascularization. Using an MCAO mouse model, we assessed the effects of pericyte‐specific *ITGA8* deletion via tamoxifen injections, followed by MCAO surgery and microvascular analysis (**Figure** **7**A). Post‐operatively, *ITGA8*iPCKO mice had lower survival rates, larger infarct sizes, and higher neurological deficit scores than controls at day 7 (Figure 7B–D). As shown in Figure 7E (upper), we detected the microvascular phenotypes in the black framed area. Histological analysis of *ITGA8*iPCKO mouse brain sections at day 7 revealed abnormal microvascular dilation and deformation in the specified region, accompanied by significant perivascular edema (Figure 7E). In *ITGA8*iPCKO; *Pdgfrb*‐tdTomato mice, post‐ischemic angiogenesis at day 7 revealed defective pericyte remodeling, manifested by disorganized pericytes with aberrant morphology and reduced coverage of neovessels (Figure 7F,G). In contrast, control mice exhibited robust pericyte recruitment and maturation, marked by elongated processes enveloping neovessels, a signature of functional maturity (Figure 7F). Although PDGFRβ+ cell density near neovessels was comparable between groups (Figure 7F,J), *ITGA8*iPCKO mice displayed disrupted pericyte‐endothelial interactions, evidenced by enlarged neovessel diameters despite unaltered vascular area, suggesting impaired BBB formation linked to *ITGA8* deficiency (Figure 7F,H,I). By day 9, peri‐infarct regions in *ITGA8*iPCKO mice similarly showed persistent pericyte dysmorphology, unchanged PDGFRβ+ cell numbers, and enlarged neovessel diameters without vascular area alterations (Figure S9A–E, Supporting Information).

Given the critical role of pericytes in BBB maintenance, we investigated the impact of *ITGA8* deficiency on post‐stroke BBB integrity. In vivo perfusion assays with tomato‐lectin revealed significantly reduced perfused vessels in peri‐infarct regions of *ITGA8*iPCKO mice at day 7, with analogous reductions observed in the infarct core despite preserved vascular area (Figure 7K; Figure S9F, Supporting Information). Concurrently, intravenous dextran‐FITC (2000 kDa) administration demonstrated more parenchymal tracer leakage in mutants, confirming BBB dysfunction (Figure 7L). Immunostaining further corroborated these findings, showing marked downregulation of tight junction proteins claudin5 and occludin, though ZO1 expression remained unaltered (Figure 7M–O). Collectively, these data establish *ITGA8* as a key regulator of pericyte‐mediated vascular remodeling and BBB stabilization after ischemic injury.

### 3.8 Rescue of BBB Function through Overexpression of *ITGA8* in Pericytes

**Figure 8:**
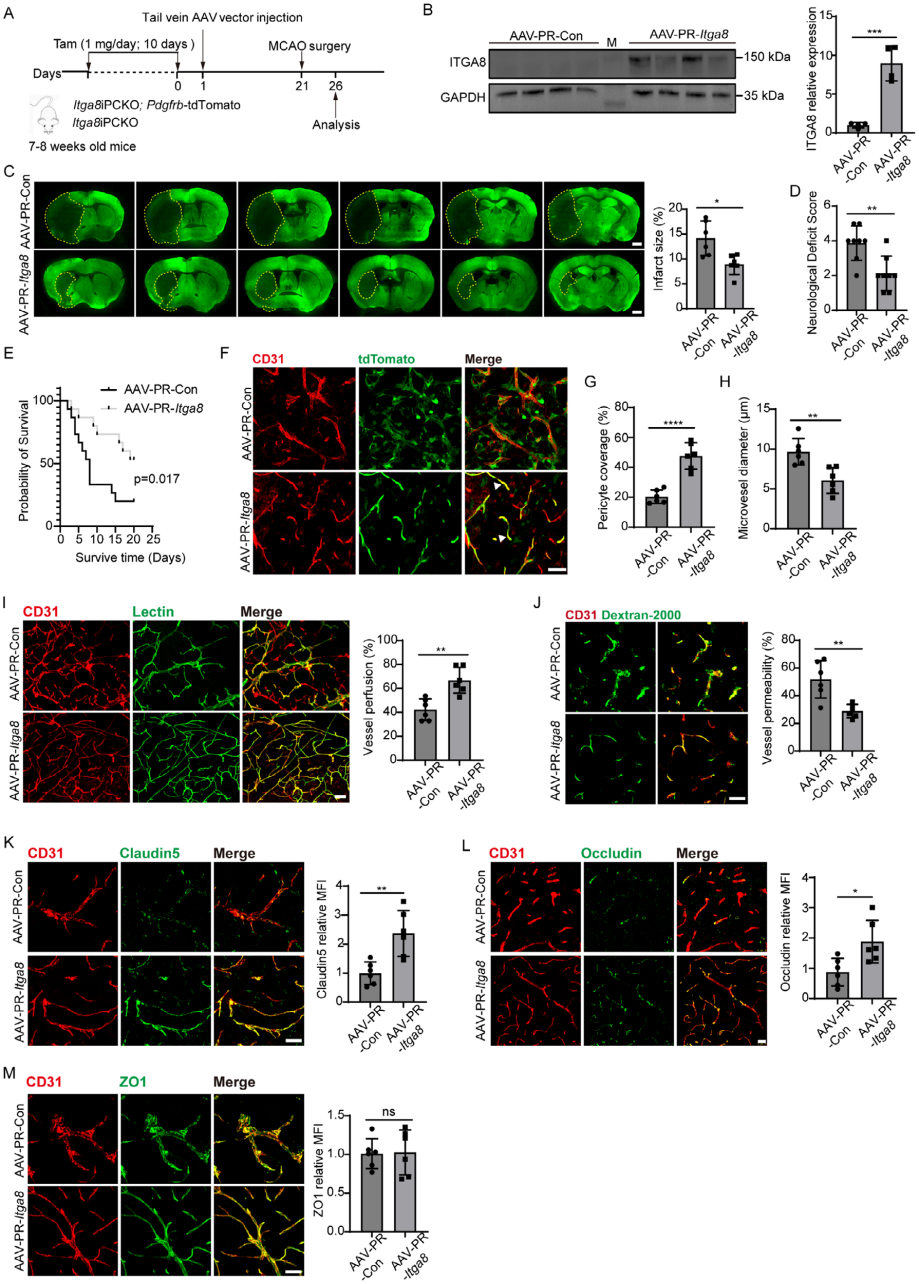
Rescue of BBB Function via *ITGA8* Overexpression in Pericytes Post‐Stroke. A) Experimental time: Tail vein injection of AAV‐PR vector to overexpress *ITGA8*, MCAO surgery and functional analysis, including neural and BBB function assessment. B) Immunoblots showing the *ITGA8* level in AAV‐PR‐con and AAV‐PR‐*ITGA8* group subjected to ischemia at 7 days. The *ITGA8* expression levels were quantified (*n* = 4 mice, *p* = 0.0004). C) MAP2 immunostaining showing the brain infarct size, the infarct area was indicated by the yellow broken line. Scale bar: 1000 µm. The infarct size of AAV‐PR‐con and AAV‐PR‐*ITGA8* group mice were quantified (*n* = 6 mice, *p* = 0.0097). D) Statistical analysis of the neurological deficit score in AAV‐PR‐con and AAV‐PR‐*ITGA8* group mice (*n* = 8 mice, *p* = 0.0033). E) Kaplan–Meier survival analysis of AAV‐PR‐con and AAV‐PR‐*ITGA8* group mice post‐MCAO (> 95%, 20 days). Values are expressed as percentage of surviving mice (*n* = 15 mice, *p* = 0.0172). F) Immunostaining of brain sections for CD31 and tdTomato showed the morphogenesis and localization of pericytes in AAV‐PR‐con and AAV‐PR‐*ITGA8* group mice. Scale bar: 20 µm. Right panels are magnified views of boxes indicated in left panels. Scale bar: 10 µm. The arrowheads showing the elongated process of pericytes and increased interconnectivity between neighboring pericytes. G,H) Quantification of pericyte coverage G) and vessel diameter H) in AAV‐PR‐con and AAV‐PR‐*ITGA8* group mice (*n* = 6 mice, *p* < 0.0001, 0.0035). I) Lectin perfusion into infarct area vessels was determined. Yellow color (green / red double‐staining) indicates blood perfused vessels. Scale bar: 50 µm. The vessel perfusion in infarct area was quantified (*n* = 6 mice, *p* = 0.0016). J) Reduced leakage of dextran‐2000 kDa from vessels in AAV‐PR‐*ITGA8* group mice mice. Scale bar: 40 µm. The vessel permeability was quantified (*n* = 6 mice, *p* = 0.0028). K–M) Immunostaining of CD31 and Claudin5 K), Occludin (), ZO1 M) in the brain sections at 26 days. Scale bar: 40 µm. The tight junctions’ MFI on the microvessels were quantified in AAV‐PR‐Con and AAV‐PR‐*ITGA8* group mice (*n* = 6 mice, *p* = 0.0034, 0.0152, 0.9093). Data represent mean ± SEM. Significance notations: ns (*p* > 0.05), ^*^ *p* < 0.05; ^**^ *p* < 0.01; ^***^ *p* < 0.001; ^****^ *p* < 0.0001. Intergroup comparisons were analyzed using unpaired 2‐tailed Student *t‐*test.

To address vascular dysfunction following ischemic stroke in *ITGA8*‐deficient mice, we investigated therapeutic *ITGA8* restoration using an adeno‐associated virus vector (AAV‐PR) targeting mural cells,^[^ ^34^ ^]^ with pericyte‐specific transduction confirmed in *Pdgfrb*‐Cre; H11‐tdTomato‐GFP reporter mice (Figure S10A, Supporting Information). AAV‐PR‐*ITGA8* was administered intravenously to *ITGA8*iPCKO mice one day post‐tamoxifen induction, followed by MCAO surgery at day 21 and functional assessments at day 26 (**Figure** **8**A). This intervention robustly upregulated *ITGA8*’s expression in ischemic area pericytes (Figure 8B), correlating with reduced infarct volume, improved neurological outcomes, and enhanced survival post‐MCAO (Figure 8C–E).

Immunofluorescence analysis revealed that *ITGA8* overexpression rescued pericyte structural deficits, promoting elongated processes and enhanced neovessel coverage compared to controls (Figure 8F,G). Reconstituted pericytes formed denser networks, normalizing peri‐infarct microvascular caliber and improving microvascular perfusion (Figure 8F,H,I). BBB integrity was restored, evidenced by reduced dextran‐FITC (2000 kDa) leakage and upregulated claudin5 / occludin expression without altering ZO1 levels (Figure 8J–M). Notably, rescued mice exhibited increased perfused vessels without vascular area alterations in infarct zones (Figure S10B, Supporting Information). These results demonstrate *ITGA8*’s critical role in post‐stroke vascular stabilization through pericyte‐mediated BBB maintenance and neovessel remodeling, highlighting its therapeutic potential for cerebrovascular repair.

## 4 Discussion

The integrity of the BBB is vital for maintaining CNS homeostasis, and pericytes are increasingly recognized as essential regulators of BBB development and repair. In this study, we identify *ITGA8*as a key pericyte‐enriched molecule that modulates BBB integrity through regulation of cytoskeletal architecture and activation of TGF‐β1 signaling. In post‐stroke neovascularization, *ITGA8* drives pericyte remodeling and neovessel maturation, with therapeutic implications for enhancing neurovascular repair following ischemic injury and potentially mitigating neurodegenerative pathologies. Collectively, we demonstrate that *ITGA8* defines a mature pericyte subpopulation critical for BBB function under both physiological and pathological conditions.

The BBB demonstrates stage‐specific integrity and transcriptional regulation during development and post‐ischemic recovery. Our investigation uncovered dynamic spatiotemporal patterns of *ITGA8* expression during BBB maturation and post‐stroke vascular remodeling. Immunohistochemical analysis revealed *ITGA8* as a mural cell‐specific marker, exhibiting distinct developmental regulation: during early neurovascular development, *ITGA8* expression localized to nascent pericyte populations associated with angiogenic sprouts. As BBB functionality progressively matured*, ITGA8*+ cells showed enhanced vascular coverage that positively correlated with barrier integrity metrics. This pericyte‐vessel association peaked in young adult mice with optimal BBB function, and then significantly declined in aged cohorts displaying characteristic barrier leakage. Following MCAO, we observed a biphasic pattern of *ITGA8* dynamics: vascular coverage initially decreased at days 1–3 post‐ischemia, followed by robust recovery reaching maximal levels by day 7–a timeline paralleling post‐stroke angiogenesis, indicating ITGA8 was reactivated. Comparative analysis with the pan‐mural cell marker PDGFRβ revealed critical spatial distinctions: in contrast to PDGFRβ+ cells, which were distributed on vessels and parenchyma, all *ITGA8*+ cells were found exclusively on vessels, suggesting a specific role in BBB function. Furthermore, these *ITGA8*‐expressing pericytes exhibited extended processes that wrapped around microvessels, forming a network with adjacent pericytes. These temporal‐spatial expression patterns raise an intriguing developmental question: Do vascular‐associated *ITGA8*+ pericytes originate from the parenchymal PDGFRβ+ cell pool? Resolution of this lineage relationship could fundamentally advance our understanding of pericyte biology in cerebrovascular homeostasis and post‐ischemic repair. We also detected a similar ITGA8 expression pattern in human brain: highest in young adults, decreased with aging, and further reduced in ischemic compared to healthy tissue. However, due to the limited sample size (*n* = 3 per group), these human data serve primarily as preliminary validation of our murine findings. Although the concordance in *ITGA8* expression supports its role in BBB integrity, larger cohorts or public dataset analyses are warranted to confirm its translational relevance. Collectively, these findings suggest ITGA8 as a maturation‐dependent regulator of BBB integrity, with its dynamic expression profile suggesting functional involvement in both developmental barrier formation and post‐stroke vascular remodeling.

To investigate the functional significance of pericyte‐expressed *ITGA8*, we generated mice with conditional pericyte‐specific deletion of *ITGA8*. These mutant mice exhibited impaired neurological function and decreased survival rates, accompanied by substantial disruption of BBB integrity. Intriguingly, while *ITGA8*‐deficient mice maintained normal pericyte numbers, we observed profound morphological alterations in these cells. Mutant pericytes displayed shortened cellular processes, diminished network complexity, and impaired endothelial contact, in vivo‐findings recapitulated in vitro through *ITGA8* knockdown or overexpression experiments in HBPCs using a pericyte‐endothelial co‐culture system. In contrast to previous studies focusing on cytokines secreted by pericytes that act on endothelial cells,^[^ ^55^ ^]^ our study offers a new perspective by highlighting the significance of pericyte morphology as a critical determinant of BBB integrity.

Our findings reveal a selective vulnerability of tight junction (TJ) components in *ITGA8*iPCKO mice, where pericyte‐specific *ITGA8* deletion preferentially disrupts transmembrane proteins Claudin‐5 and Occludin while sparing the cytoplasmic adaptor ZO‐1, suggesting distinct maintenance mechanisms between transmembrane TJ elements and their cytoskeletal anchors. This dichotomy likely stems from differential regulatory pathways: Claudin‐5 and Occludin are modulated by integrin‐sensitive transcriptional programs (PI3K/Akt and Wnt/β‐catenin), whereas ZO‐1 stability depends on post‐translational mechanisms through cytoskeletal tethering.^[^ ^56^, ^57^, ^58^ ^]^ The preserved ZO‐1 architecture implies that pericyte‐derived *ITGA8* signaling specifically sustains membrane‐embedded TJ components rather than global junctional complexes. Our results delineate a precision mechanism of pericyte‐mediated blood‐brain barrier support, where ITGA8 activity selectively fortifies the transmembrane sealing apparatus essential for paracellular barrier function, independent of intracellular scaffold maintenance.

To accurately evaluate ITGA8 function, we employed validated pericyte markers and complementary experimental models. CD13 and PDGFRβ were chosen based on their robust expression in brain pericytes and widespread use in cerebrovascular studies. While neither is pericyte‐exclusive, their co‐labeling, combined with anatomical localization, allowed us to distinguish pericytes from smooth muscle cells and fibroblasts in both tissue sections and cultured cells. In vitro, we utilized a pericyte–endothelial co‐culture model that recapitulates essential BBB features, enabling precise analysis of pericyte morphology, junctional organization, and response to pathway modulation (e.g., ROCK inhibition, TGF‐β1 supplementation). In vivo, we used a transient MCAO stroke model to confirm *ITGA8*’s functional relevance within the full neurovascular unit. The parallel use of reductionist and physiological systems ensured mechanistic insight and translational validity, thereby directly addressing concerns regarding specificity, cellular identity, and relevance to cerebrovascular injury.

Integrin α8β1 engagement is known to connect the extracellular matrix to the intracellular actin cytoskeleton,^[^ ^59^, ^60^ ^]^ activating Rho GTPase pathways that govern cell shape and contractility.^[^ ^61^ ^]^ In our models, *ITGA8* loss‐of‐function in pericytes resulted in blunted RhoA–ROCK signaling, evidenced by disorganized actin and fewer contractile stress fibers. Pericytes lacking *ITGA8* failed to properly elongate and wrap around endothelial tubes, instead adopting a retracted morphology with diminished processes. These cytoskeletal defects were ameliorated by pharmacological ROCK inhibition, implicating excessive RhoA–ROCK activity in the absence of normal *ITGA8* signaling. Indeed, Rho/ROCK pathways are well known to regulate pericyte shape and motility, our data suggest ITGA8 serves as an upstream modulator of this pathway, anchoring pericytes to the matrix and promoting the actomyosin‐driven tension necessary for maintaining vessel coverage. In parallel, *ITGA8* emerged as a mediator of TGF‐β1 activation in the neurovascular unit. We found that pericyte‐specific *ITGA8* influences the bioavailability of active TGF‐β1, a cytokine crucial for vessel maturation and BBB integrity. In *ITGA8*‐deficient conditions, TGF‐β1 signaling was attenuated, while restoring *ITGA8* function enhanced TGF‐β1 activation. In the cerebral microvasculature, TGF‐β1 signaling orchestrated by pericytes and integrins is a known determinant of angiogenic quiescence and BBB phenotype,^[^ ^62^ ^]^ our findings add *ITGA8* to this paradigm, suggesting that pericytes use *ITGA8* to mechanically transmit signals that activate TGF‐β1, thereby reinforcing endothelial tight junctions and basement membrane deposition. Together, the Rho/ROCK and TGF‐β1 pathways provide a dual mechanism whereby *ITGA8* regulates pericyte morphology and paracrine signaling.

In this study, although the RGD peptide was utilized in our in vitro experiments to inhibit the interaction between *ITGA8* and TGFβ—resulting in a significant disruption of vascular integrity—we acknowledge an important limitation regarding its specificity. The RGD motif is known to be recognized by multiple integrins beyond ITGA8, such as αvβ3, αvβ5, and α5β1, which are also involved in cell adhesion and ECM signaling. Thus, the observed effects may not be solely attributable to the blockade of ITGA8–TGFβ engagement, but could also reflect unintended inhibition of other RGD‐dependent pathways. To overcome this, future studies should employ *ITGA8*‐specific function‐blocking antibodies, thereby reducing off‐target effects and firmly establishing the non‐redundant role of ITGA8–TGFβ signaling in vascular function.

Cerebrovascular integrity is a critical determinant of stroke outcome–compromised BBB function contributes to edema, hemorrhagic transformation, and prolonged neuroinflammation.^[^ ^63^, ^64^ ^]^ Yet, current acute stroke therapies (e.g., thrombolysis or thrombectomy) do not directly address vascular repair. Our findings suggest that augmenting *ITGA8*‐mediated signaling in pericytes could be a novel strategy to stabilize the BBB in the post‐ischemic period. By strengthening pericyte attachment and promoting TGF‐β1 activation, ITGA8 activity might hasten the re‐sealing of the BBB, thereby reducing secondary brain injury. Notably, pericytes respond very early to ischemic insult, altering their behavior before overt endothelial damage occurs. Harnessing this early response through *ITGA8* could confer a timely reinforcement of the vasculature. For instance, pharmacological activation of *ITGA8* signaling or administration of *ITGA8* ligands (such as a nephronectin‐derived peptide or an RGD‐mimetic that specifically engages integrin α8β1) might enhance pericyte‐led vessel repair. An alternative approach could be gene therapy or small molecules to upregulate *ITGA8* expression in pericytes during the subacute phase of stroke. Importantly, any *ITGA8*‐targeted intervention would need to be finely tuned: while enhancing *ITGA8* function might promote BBB closure and neuroprotection, one must also consider the context of TGF‐β1 signaling to avoid excess fibrosis or gliosis.

This study reveals the critical role of *ITGA8* in maintaining BBB integrity and promoting pericyte‐mediated vascular maturation, offering new therapeutic insights for neurodegenerative disorders such as Alzheimer's disease (AD) and multiple sclerosis (MS). In AD, *ITGA8* may help reduce Aβ deposition and neuroinflammation; in MS, it could enhance vascular stability and suppress immune cell infiltration. Moreover, the ability of *ITGA8* to modulate pericyte function may also extend to other neurological conditions involving BBB impairment, such as Parkinson's disease. Targeting *ITGA8* holds promise as a universal strategy for promoting neurovascular repair and delaying disease progression. Future studies should focus on developing *ITGA8*‐based interventions and validating their efficacy in aging and neurodegenerative models.

In summary, this study uncovers *ITGA8* as a critical regulator of pericyte morphology and BBB integrity, with distinct effects on junctional protein organization and signaling pathways like Rho/ROCK and TGF‐β1. *ITGA8*‐expressing pericytes emerge as key players in vascular development and in the endogenous repair response after stroke. The insights gained not only advance our understanding of pericyte biology in cerebrovascular health and disease but also open the door to targeting the pericyte–matrix interface in therapeutic strategies.

## Conflict of Interest

The authors declare no conflict of interest.

## Supporting information



Supporting Information

## Data Availability

The data that support the findings of this study are available from the corresponding author upon reasonable request.
