# Supplementary material for: Integrin α8‐Mediated Pericyte Morphogenesis Controls Blood‐Brain Barrier Integrity
Source: Adv Sci (Weinh). 2025 Oct 14;12(48):e15374. doi: 10.1002/advs.202415374 (PMC12752560; doi:10.1002/advs.202415374)
Supplement: Supplementary file 1 — Supporting Information [file ADVS-12-e15374-s001.docx]

Supplementary figures and figure legends

Figure S1


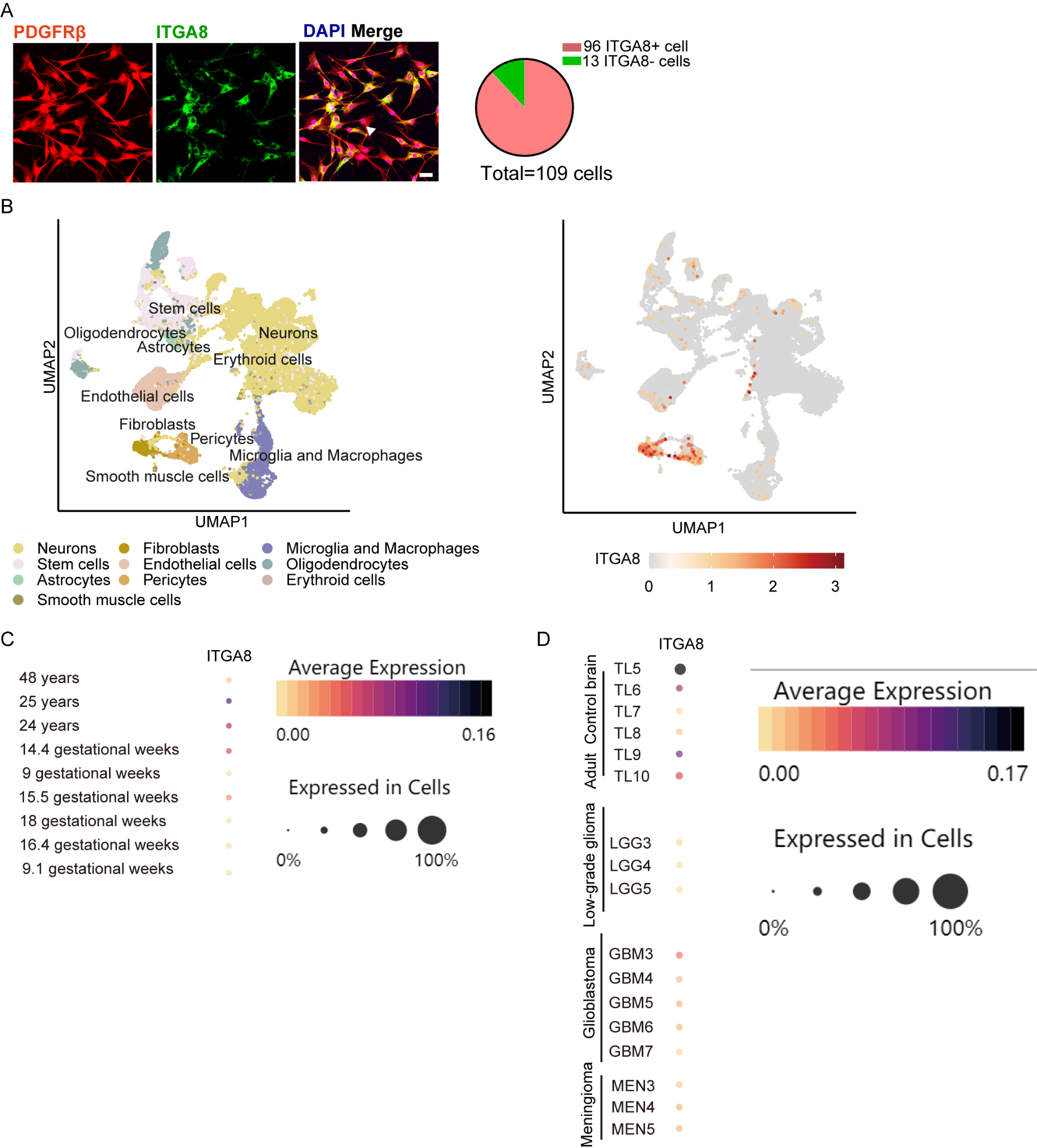


**Figure S1. Characterization of ITGA8 Expression in PDGFRβ+ Cells**

1. Double immunostaining of human brain primary pericytes (HBPCs), with PDGFRβ stained in red and ITGA8 in green. Scale bar, 40μm. Quantitation of the number of *Itga8*-expressing cells in PDGFRβ+ cells (n = 3 independent experiments).
2. UMAP plots of total human brain cells derived from fetus (https://waelchli-lab-human-brain-vasculature-atlas.ethz.ch), revealing 10 major clusters. Each point in the plot represents an individual cell, indicated by a droplet barcode and assigned a color based on its cluster. Analysis of ITGA8 expression across all cells on UMAP plots.

(C-D) The graph showing the expression of ITGA8 at different development stage and different pathological conditions, which were analyzed from the single-cell sequencing data (<https://brain-vasc.cells.ucsc.edu>).

Figure S2


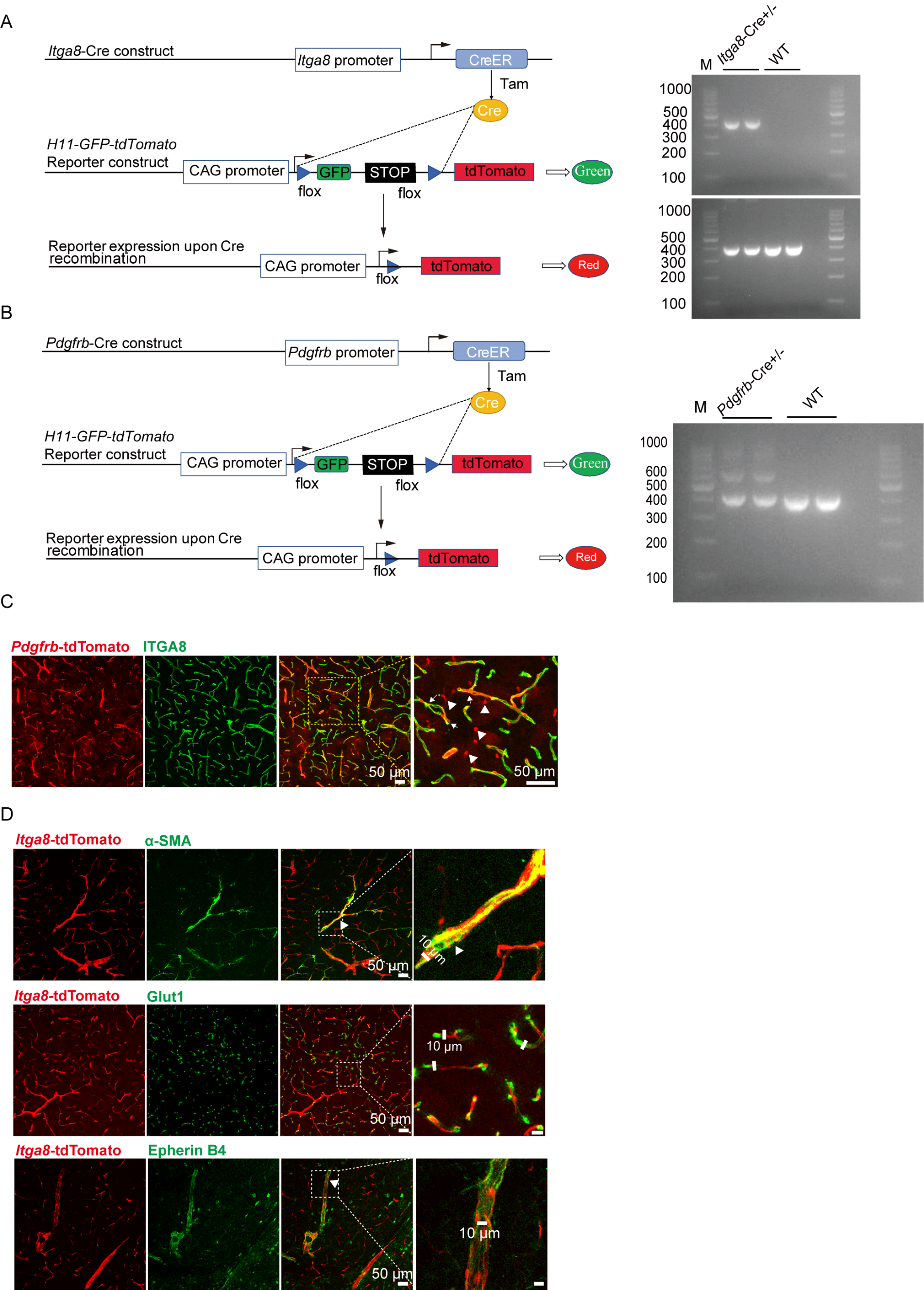


**Figure S2.** **Characterization of Reporter Mice and Analysis of ITGA8 Expression Patterns**

1. Genetic engineering for *Itga8*-expressing cell labeling. (Left) the structure of the *Itga8*-Cre and H11-GFP-tdTomato transgenes, alongside a schematic of the *H11-GFP-tdTomato* construct pre and post Cre-mediated recombination. In *Itga8*-Cre mice, Cre recombinase is expressed. The *H11-GFP-tdTomato* transgene contains a CAG promoter driving a floxed ZsGreen sequence. Cre-mediated recombination excises ZsGreen, enabling the promoter to drive tdTomato expression in ITGA8+ cells. (Right) the results of PCR analysis conducted on genomic DNA for genotyping to confirm the presence of the *Itga8*-Cre allele.

(B) (Left) Structure of *Pdgfrb*-Cre and H11-GFP-tdTomato transgenes, along with a schematic representation of the H11-GFP-tdTomato construct before and after Cre-mediated recombination. In *Pdgfrb*-Cre mice, the expression of Cre recombinase leads to the excision of the ZsGreen sequence, subsequently allowing the CAG promoter to drive tdTomato expression specifically in PDGFRβ+ cells. (Right) PCR analysis of genomic DNA for genotyping to verify the presence of the *Pdgfrb-*Cre allele.

(C) The immunofluorescence staining of brain sections from *Pdgfrb*-Cre; H11-GFP-tdTomato mice, showing the ITGA8 expression was within the PDGFRβ+ cells. Arrowheads indicate the scattered PDGFRβ+ cells in the parenchyma. Scale bar, 40 μm.

(D) Differential identification of brain vasculature in *Itga8*-Cre; H11-GFP-tdTomato mice using α-SMA (arterioles), Glut1 (capillaries / BBB), and Ephrin B4 (venules) immunostaining with vessel diameter criteria. (Capillary diameter < 10 μm).

Figure S3


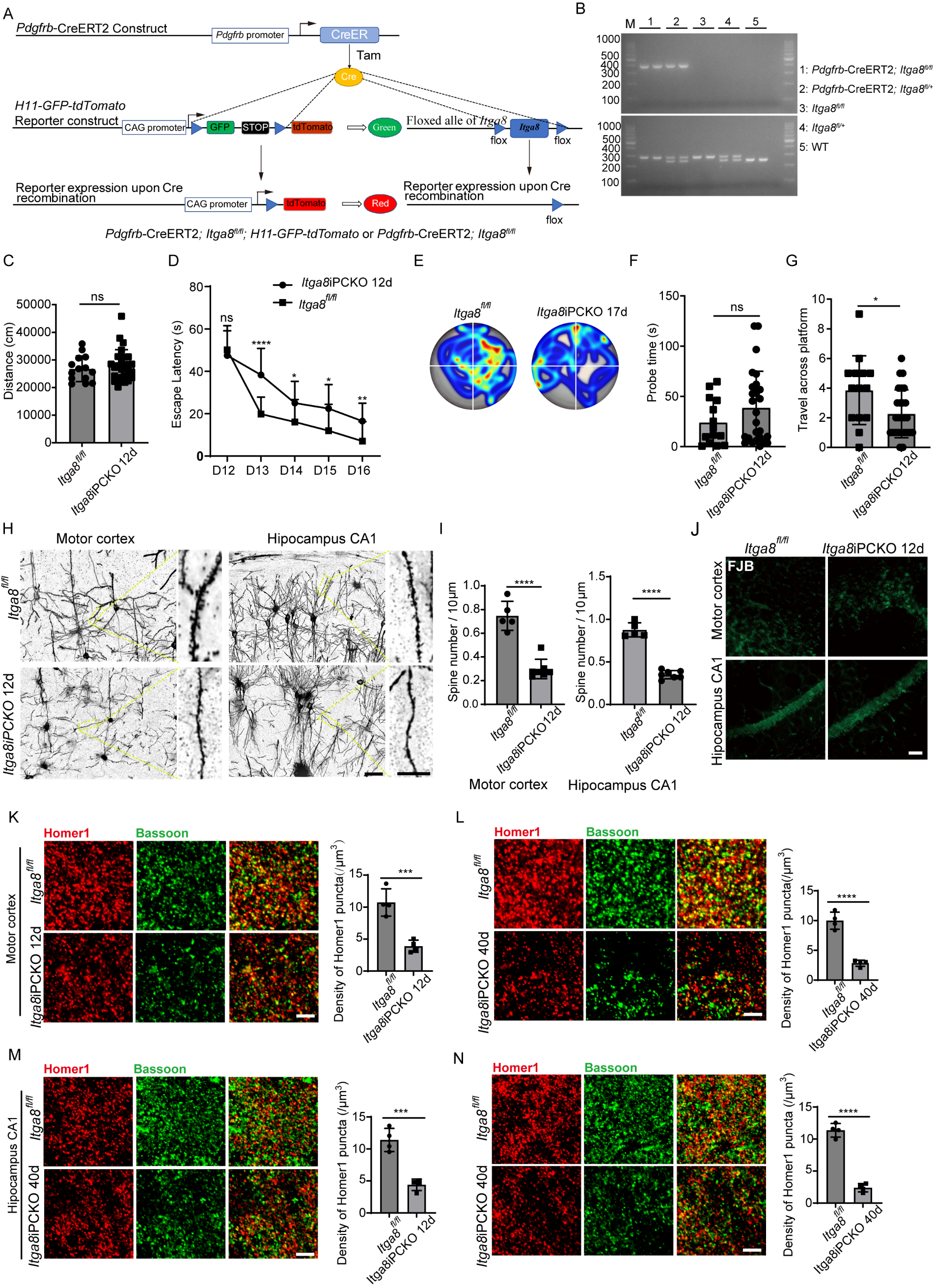


**Figure S3 Impact of Pericyte *Itga8* Deletion on Neural Function**

1. Schematic Representation of *Pdgfrb*-ERT-Cre, H11-GFP-tdTomato, and *Itga8^fl/fl^* Transgenes: This diagram illustrates the structure and functionality of the *Pdgfrb-*ERT-Cre and H11-GFP-tdTomato transgenes alongside the *Itga8* floxed allele (*Itga8^fl/fl^*) before and after Cre-mediated recombination. In *Pdgfrb*-ERT-Cre mice, a tamoxifen-activated Cre-ERT2 recombinase facilitates the excision of either the ZsGreen sequence or the Itga8 gene. This action allows the CAG promoter to either drive the expression of tdTomato or effectively knock out *Itga8* in PDGFRβ+ cells.
2. PCR analysis for genotyping to detect *Pdgfrb-*CreERT and *Itga8^fl/fl^* alleles in mice.
3. Measurement of initial swimming distances in the MWM test before training trials at 12 days. Data from *Itga8^fl/fl^* (n = 14 mice) and *Itga8*iPCKO (n = 26 mice), *p* = 0.5132.
4. The acquisition trial was performed for consecutive five days. Escape latency comparison during the acquisition phase of the MWM test at various time points. Data from *Itga8^fl/fl^* (n = 14 mice) and *Itga8*iPCKO (n = 26 mice), *p* = < 0.4986, 0.0001, 0.0132, 0.0112, 0.0018.
5. Representative swimming traces during the probe trial at 17 days in the MWM test.

(F-G) Quantification of platform zone crossings (G) and latency to first reach the platform zone (F) during the probe trial at 17 days in the MWM test. Data from *Itga8^fl/fl^* (n = 14 mice) and *Itga8*iPCKO (n = 26 mice), *p* = 0.1832, 0.0147.

(H) Golgi staining of pyramidal neurons in the motor cortex and hippocampus CA1 at 12 days. Scale bar, 50 μm. Higher magnification images of dendritic spines are shown in the right panels. Scale bar, 15 μm.

(I) Quantitative analysis of dendritic spine density in neurons of the motor cortex and hippocampus CA1 at 12 days. *Itga8*iPCKO (n = 7 mice), *Itga8^fl/fl^* (n = 5 mice), all *p* < 0.0001.

(J) FJB staining of brain sections showing no signs of neuronal degeneration in *Itga8*iPCKO mice at 12 days. Scale bar, 30 μm.

(K-N) Representative images of Homer1 and Bassoon expression in motor cortex and hippocampus CA1 region in *Itga8*iPCKO mice and *Itga8^fl/fl^* mice at 12 days or 40 days. Scale bar, 5μm. The density of Homer1 puncta was quantified (n = 4 mice, *p* = < 0.0012, 0.0001, 0.0004, 0.0001).

Data represent mean ± SEM. Significance notations: ns (*p* > 0.05), **p* < 0.05; ***p* < 0.01; ****p* < 0.001; *****p* < 0.0001. Intergroup comparisons were analyzed using unpaired 2-tailed Student *t* test.

Figure S4


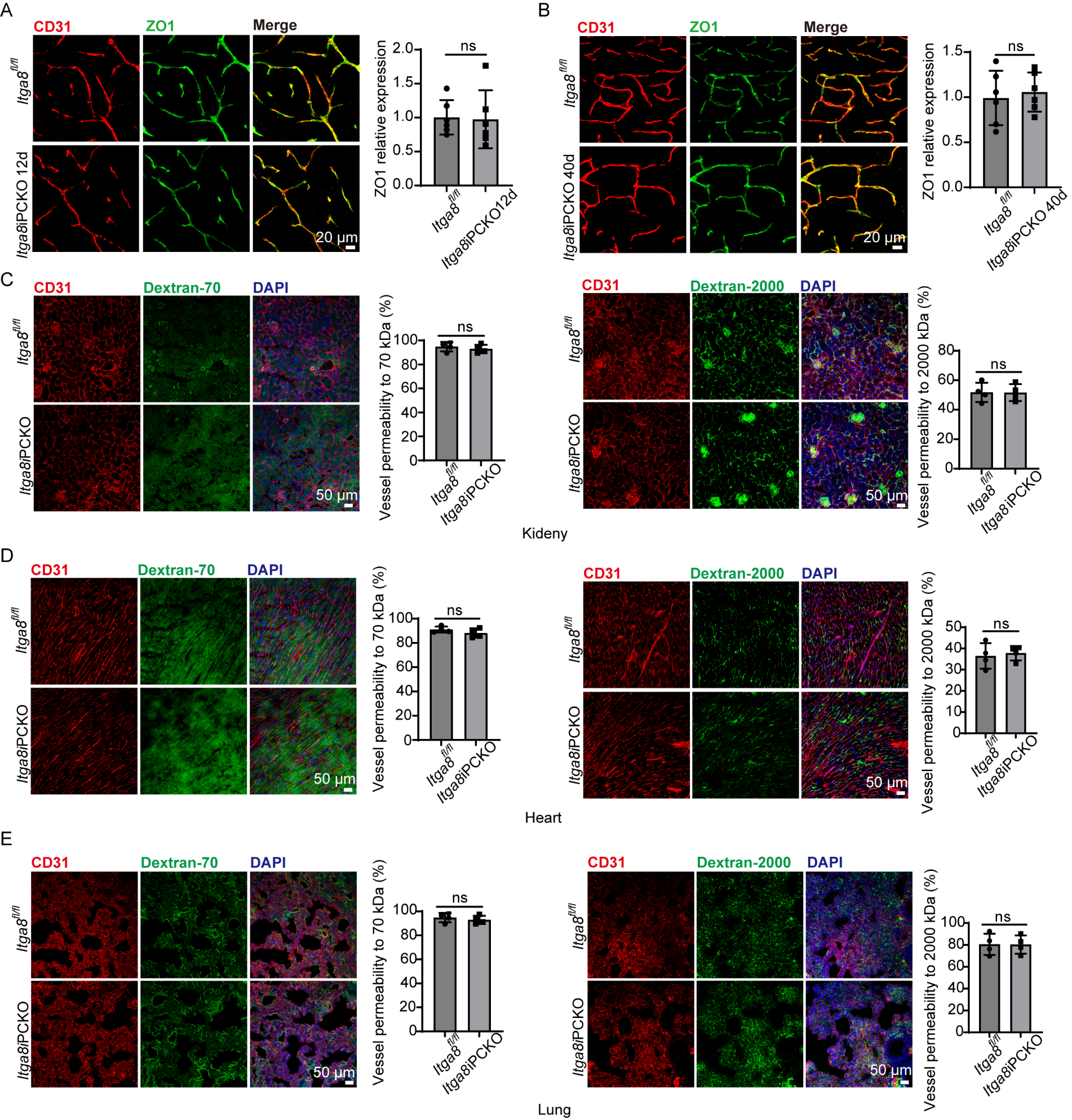


**Figure S4. Expression of ZO1 and Microvascular Permeability in *Itga8*iPCKO and *Itga8^fl/fl^* Mice**

(A-B) CD31 and ZO1 double immunostaining of brain sections at 12 days (A) and 40 days (B). Quantification of ZO1 relative mean fluorescence intensity (MFI) on CD31+ vessels at 12 days (n = 6 mice, *p* = 0.8937, 0.6742).

(C-E) *In vivo* tail vein injection of Dextran-70 or 2000 kDa to determine the integrity of microvessels in kideny (C), heart (D), and lung (E) in *Itga8*iPCKO mice at 40 days (n = 4, *p* = 0.4888, 0.9793, 0.2640, 0.7249, 0.7947, 0.9744).

Data represent mean ± SEM. Significance notations: ns (*p* > 0.05). Intergroup comparisons were analyzed using unpaired 2-tailed Student *t* test.

Figure S5


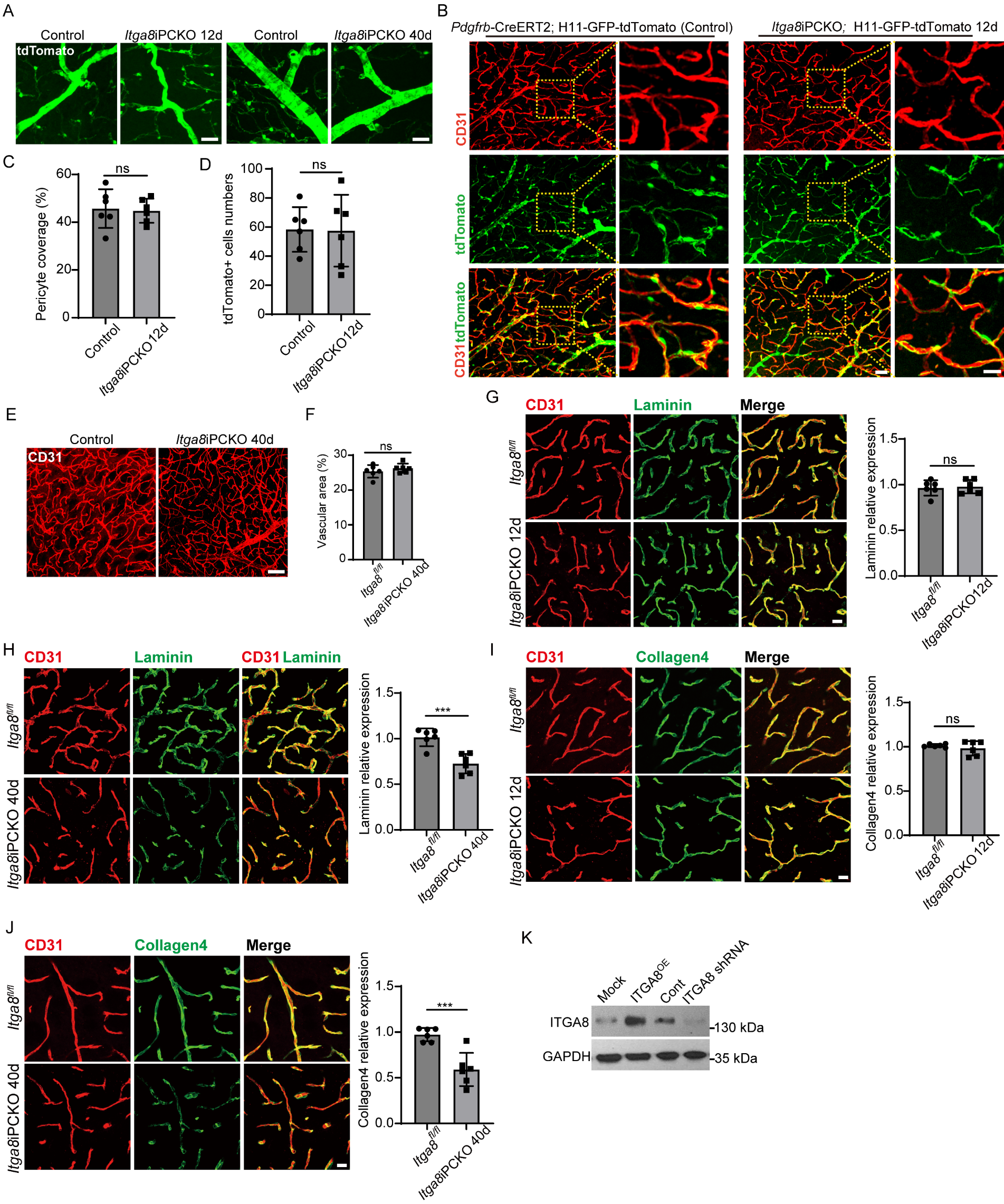


**Figure S5 Characterization of Brain Vessel Phenotypes Following *Itga8* Deletion**

1. Representative images of tdTomato+ smooth muscle cells on arterioles at 12 days and 40 days, showing no changes in smooth muscle cells coverage and morphology between *Itga8*iPCKO and control mice. Scale bar, 30 μm.
2. Immunostained brain sections of of *Itga8*iPCKO; *Pdgfrb-*tdTomato mice and control mice at 12 days for vessels. Scale bar, 40 μm. Higher magnification images in right panels, indicated by yellow boxes. Scale bar, 20 μm.

(C-D) Quantification of pericyte coverage (C) and pericytes number (D) in brain sections at 12 days (n = 6 mice, *p* = 0.8223, 0.9452).

(E-F) Immunostaining of CD31 showing brain vasculature of *Itga8*iPCKO mice *and Itga8^fl/fl^* mice at 40 days (E). Scale car, 75 μm. Quantification of brain vascular area (F) (n = 6 mice, *p* = 0.3541).

(G-H) Double immunostaining for CD31 and Laminin in brain sections at 12 days (G) and 40 days (H). Scale bar, 20 μm. Quantification of Laminin relative MFI on CD31+ vessels in brain sections at 12 days (n = 6 mice, *p* = 0.7488, 0.0006).

(I-J) Double immunostaining for CD31 and Collagen4 in brain sections at 12 days (I) and 40 days (J). Scale bar, 20 μm. Quantification of Collagen4 relative MFI on CD31+ vessels in brain sections at 12 days (n = 6 mice, *p* = 4012, 0.0008).

(K)Western blot analysis demonstrating ITGA8 overexpression using Lentivirus-hITGA8 and its knockdown using Lentivirus-hITGA8-shRNA (n = 3 independent experiments).

Data represent mean ± SEM. Significance notations: ns (*p* > 0.05); ****p* < 0.001. Intergroup comparisons were analyzed using unpaired 2-tailed Student *t* test.

Figure S6


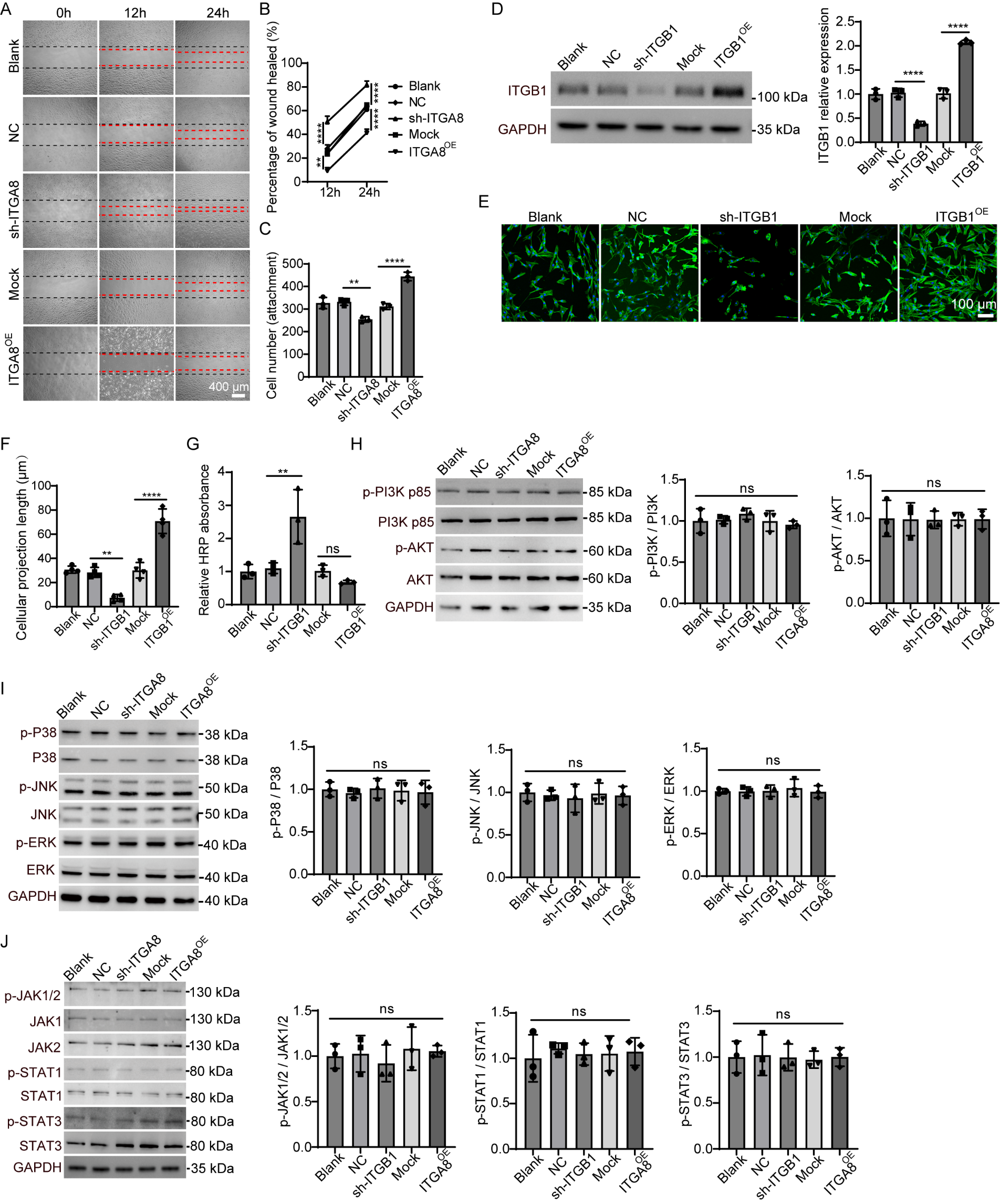


**Figure S6. ITGA8-TGFβ1 Signaling Regulates Pericytes Function**

(A-B) Wound-healing test was used to detect HBPCs migration after ITGA8 knockdown or overexpression. The relative distance of cell migration was quantified (n = 3 independent experiments, all *p* < 0.0001).

(C) Cell adhesion assay was used to examine HBPCs adhesion ability after ITGA8 knockdown or overexpression (n = 3 independent experiments, *p* = < 0.0022, 0.0001).

(D) ITGB1 modulation in pericytes via lentiviral-mediated shRNA knockdown and cDNA overexpression demonstrated by western blotting (n = 3 independent experiments, all *p* < 0.0001).

(E) Immunostaining of Phalloidin revealed the intracellular stress fibers and cytoskeleton changes in pericytes following manipulation of ITGB1 expression.

(F) Quantification of length of pericyte cellular projections following manipulation of ITGB1 expression (n = 3 independent experiments, *p* = < 0.0013, 0.0001).

(G) Endothelial barrier integrity assessment in transwell co-culture system with ITGB1-modulated pericytes (knockdown / overexpression) (n = 3 independent experiments, *p* = 0.0048, 0.8183).

(H) Western blot analysis showing the expression of PI3K-AKT signaling proteins following manipulation of ITGA8 expression (n = 3 independent experiments, *p* = > 0.5799, 0.9999).

(I) Western blot analysis showing the expression of MAPK signaling proteins following manipulation of ITGA8 expression (n = 3 independent experiments, *p* = 0.9507, 0.9581, 0.9355).

(J) Western blot analysis showing the expression of JAK-STAT signaling proteins following manipulation of ITGA8 expression (n = 3 independent experiments, *p* = 0.8316, 0.9521, 0.9967).

Data represent mean ± SEM. Significance notations: ns (*p* > 0.05), ***p* < 0.01, *****p* < 0.0001; Comparisons between multiple groups were made using one-way ANOVA test followed by Tukey’s HSD post hoc test.

Figure S7


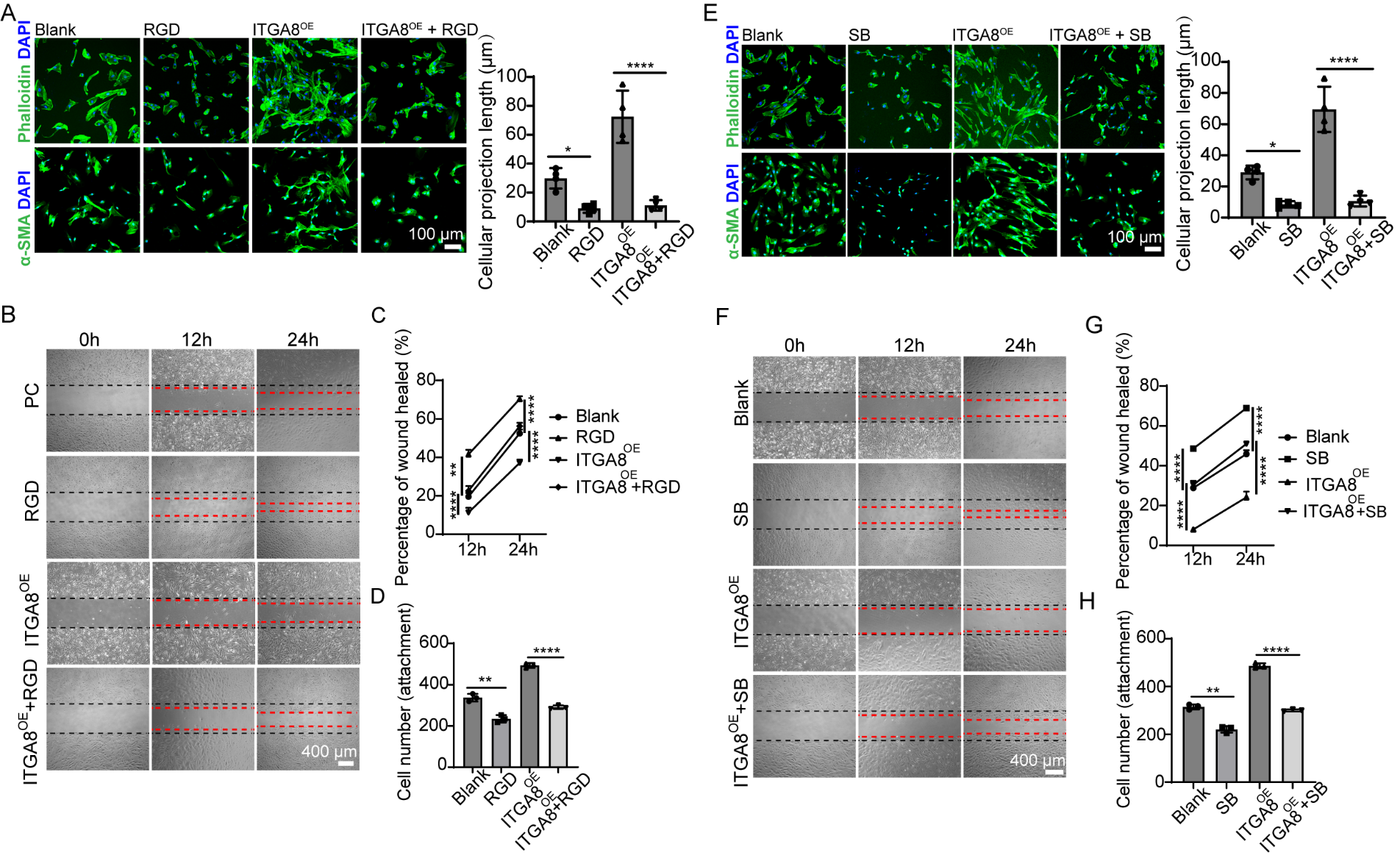


**Figure S7. ITGA8-TGFβ1 Signaling Regulates Pericytes Morphogenesis**

1. Immunostaining of Phalloidin and α-SMA revealed the intracellular stress fibers and cytoskeleton changes in pericytes following manipulation of ITGA8 expression or treatment with RGD peptide (n = 3 independent experiments, *p* = < 0.0428, 0.0001).

(B-C) Wound-healing test was used to detect HBPCs migration after ITGA8 overexpression or treatment with RGD peptide (B). The relative distance of cell migration was quantified (C) (n = 3 independent experiments, *p* = < 0.0017, 0.0001, 0.0001, 0.0001).

(D) Cell adhesion assay was used to examine HBPCs adhesion ability after ITGA8 overexpression or treatment with RGD peptide (n = 3 independent experiments, *p* = < 0.0035, 0.0001).

(E) Immunostaining of Phalloidin and α-SMA revealed the intracellular stress fibers and cytoskeleton changes in HBPCs following manipulation of ITGA8 expression or treatment with SB431542 (n = 3 independent experiments, *p* = < 0.0145, 0.0001).

(F-G) Wound-healing test was used to detect HBPCs migration after ITGA8 overexpression or treatment with SB431542 (F). The relative distance of cell migration was quantified (G) (n = 3 independent experiments, all *p* < 0.0001).

(H) Cell adhesion assay was used to examine HBPCs adhesion ability after ITGA8 overexpression or treatment with SB431542 (n = 3 independent experiments, *p* = < 0.0042, 0.0001).

Data represent mean ± SEM. Significance notations: ns (*p* > 0.05), **p* < 0.05, ***p* < 0.01, *****p* < 0.0001; Comparisons between multiple groups were made using one-way ANOVA test followed by Tukey’s HSD post hoc test.

Figure S8


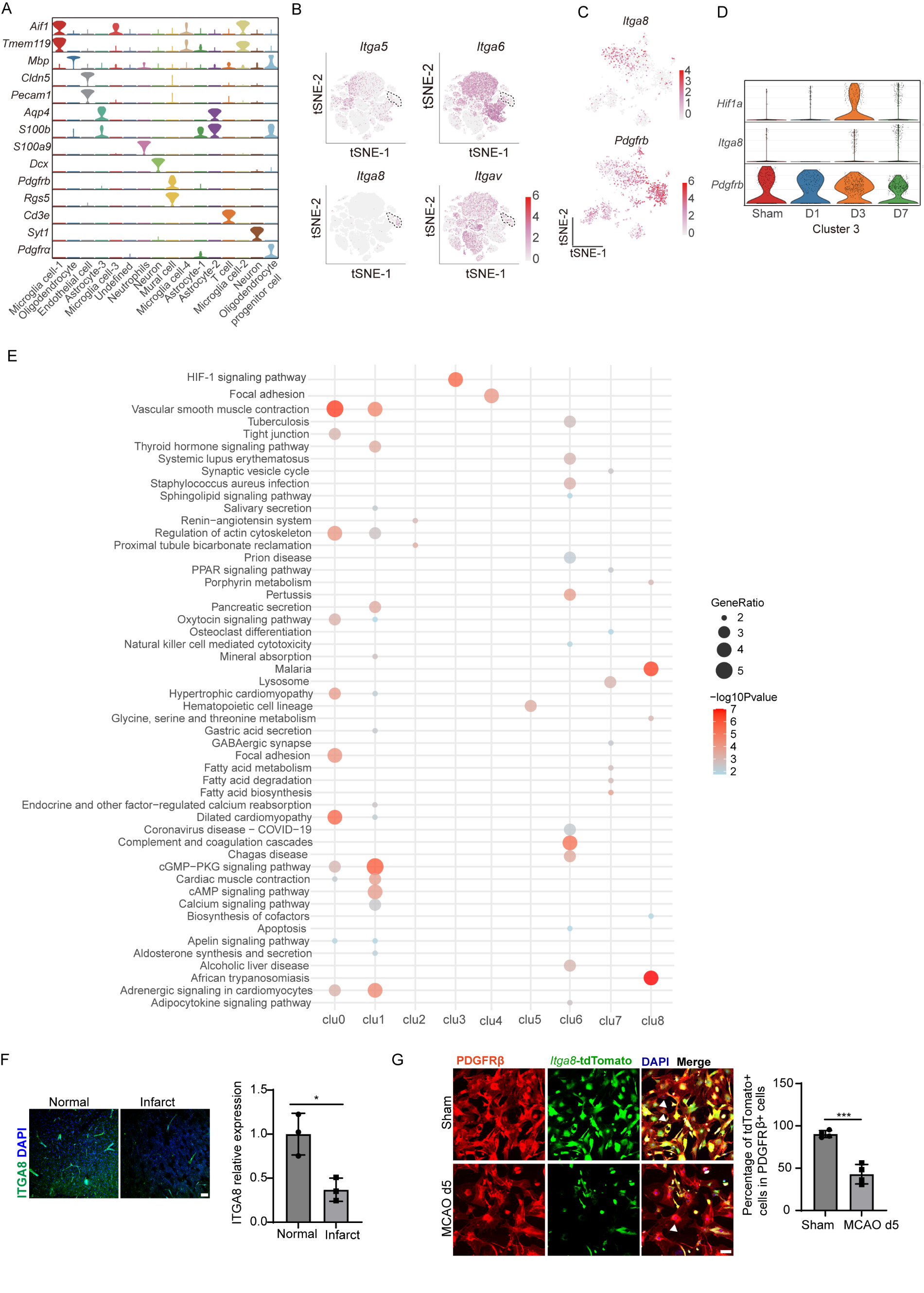


**Figure S8 Sc-RNA Sequencing of Brain Tissues Post-MCAO**

1. Violin plots display the expression of top cell-type specific marker genes across each identified cluster.
2. Comparative expression analysis of *Itga8* with other integrins (*Itga5*, *Itga6*, *Itgav*) across all cells on tSNE plots.

(C) Expression of *Pdgfrb* and *Itga8* projected onto tSNE plots in various clusters of mural cells.

(D) Analysis of the expression patterns of the *Hif1*, *Itga8*, and *Pdgfrb* genes, with a notable increase in *Hif1* expression within cluster 3 at day 3 post-ischemia.

(E) KEGG signaling pathway analysis in different clusters of mural cells.

(F) ITGA8 expression was analyzed by immunostaining in human infarcted brain sections from stroke patients and quantified (n = 3 samples, *p* = 0.0156).

(G) PDGFRβ immunostaining of PDGFRβ+ cells collected via FACS from *Itga8*-Cre; H11-GFP-tdTomato mice at post-stroke day 5. Scale bar, 40μm. Quantitation of the percentage of *Itga8*-expressing cells in PDGFRβ+ cells (n = 4 mice, *p* = 0.0002).

Data represent mean ± SEM. Significance notations: ****p* < 0.001. Intergroup comparisons were analyzed using unpaired 2-tailed Student *t* test.

Figure S9


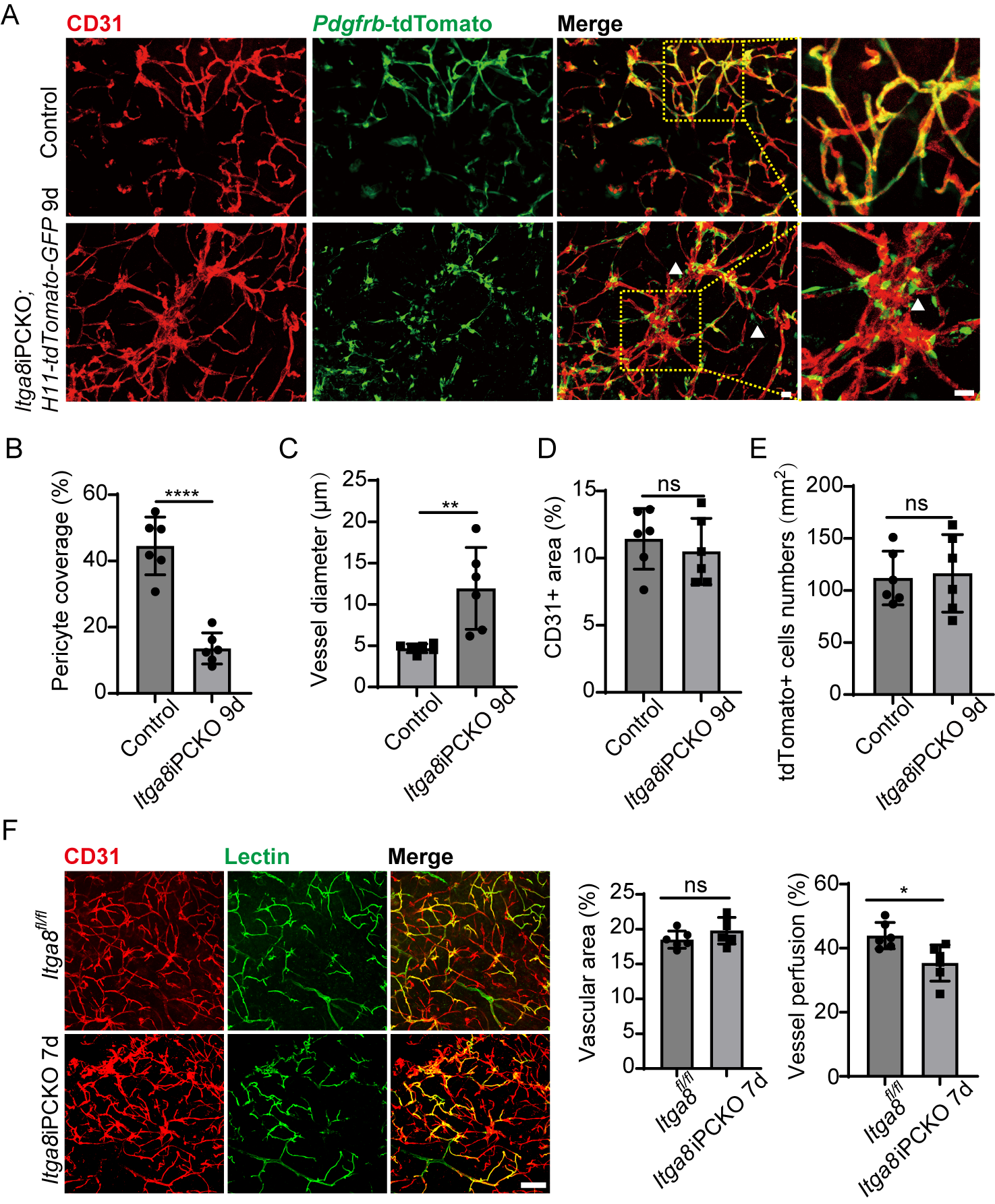


**Figure S9 Analysis of Neovascularization and Pericyte Alterations in *Itga8*iPCKO Mice**

1. Brain sections of *Itga8*iPCKO*; H11-GFP-tdTomato* mice and control mice at 9 days immunostained for vessels. Scale bar, 20 μm. Yellow boxes correspond to higher magnification images shown in right panel. Scale bar, 20 μm. Arrowheads showing the abnormal pericyte shape and poor association between pericytes and endothelial cells.

(B-E) Quantification of tdTomato+ pericyte coverage on vessels (B), vessel diameter (C), vascular area (D) and PDGFRβ+ cells number (E) at 9 days in *Itga8*iPCKO mice and control mice (n = 6 mice, *p* = < 0.0001, 0.0051, 0.5089, 0.8123).

1. Representative images of vessel perfusion in the infarct area. Scale bar, 40 μm. The vascular and percentage of perfused vessels were quantified (n = 6 mice, *p* = 0.1921, 0.0132).

Data represent mean ± SEM. Significance notations: ns (*p* > 0.05), **p* < 0.05 ***p* < 0.01, *****p* < 0.0001. Intergroup comparisons were analyzed using unpaired 2-tailed Student *t* test.

Figure S10


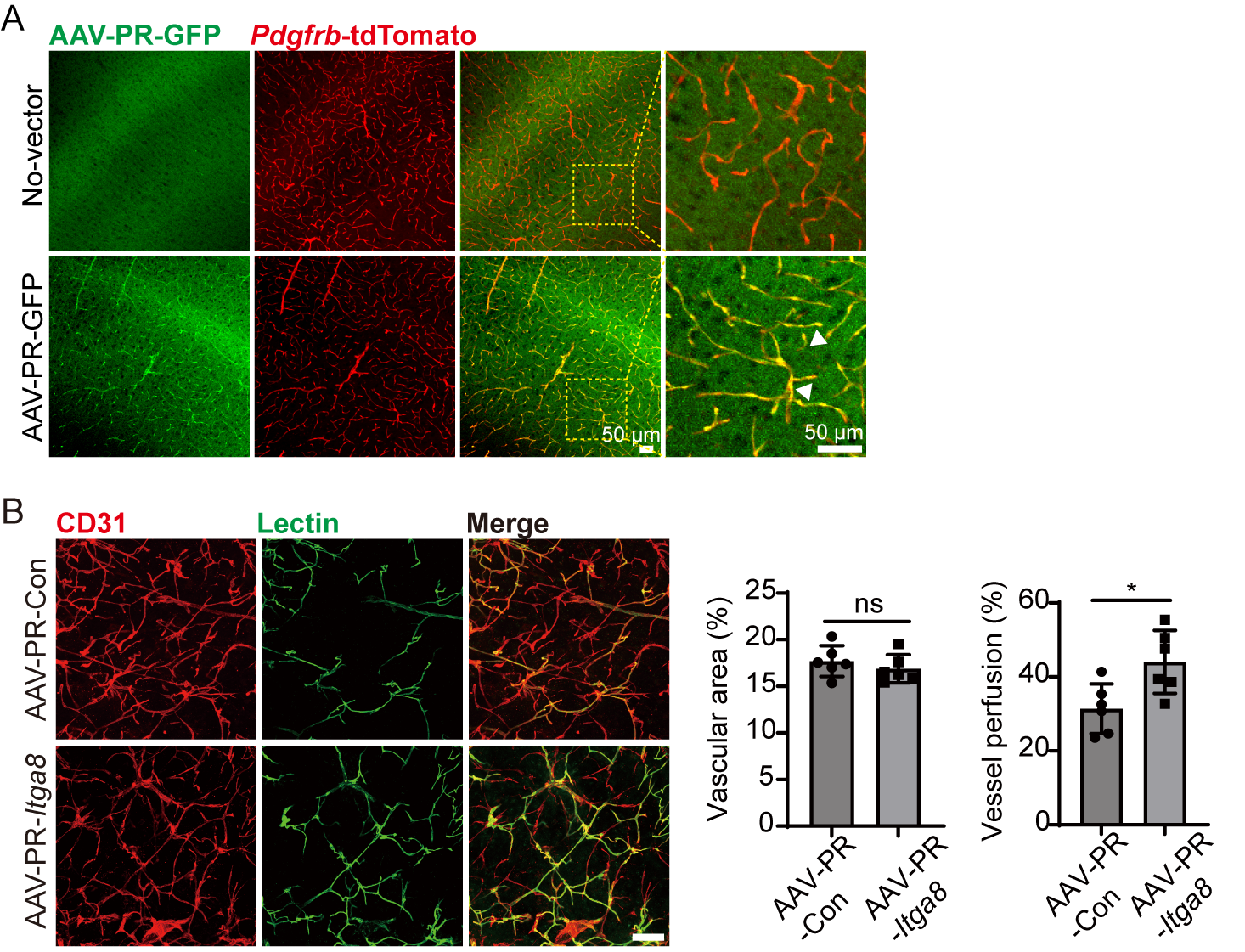


**Figure S10 Overexpression of *Itga8* in Pericytes Increased the Perfused Vessels in Infarct Core Area.**

(A) Confocal micrographs demonstrate AAV-PR-GFP-mediated transduction in mouse brain mural cells, with arrowheads highlighting coordinate GFP / tdTomato fluorescence in PDGFRβ+ cells (n = 3 independent experiments).

(B) Representative images of vessel perfusion in the infarct area of AAV-PR-*Itga8* and AAV-PR-Con group mice. Scale bar, 40 μm. The percentage of perfused vessels was quantified (n = 6 mice, *p* = 0.3891, 0.0169).

Data represent mean ± SEM. Significance notations: ns (*p* > 0.05), **p* < 0.05. Intergroup comparisons were analyzed using unpaired 2-tailed Student *t* test.
